# Medicinal flora of the baiku yao people — An ethnobotanical documentation in South China

**DOI:** 10.1186/s12906-024-04545-8

**Published:** 2024-06-21

**Authors:** Tingting Li, Binsheng Luo, Yuanming Tong, Guiyuan Wei, Ling Chai, Renchuan Hu

**Affiliations:** 1 Jiangbin Hospital of Guangxi Zhuang Autonomous Region, Nanning, 530000 China; 2 Lushan Botanical Garden, Jiangxi Province and Chinese Academy of Sciences, Lushan, 332900 China; 3 Guangxi Key Laboratory of Traditional Chinese Medicine Quality Standards, Guangxi Institute of Chinese Medicine & Pharmaceutical Science, Nanning, 530022 China

**Keywords:** Baiku Yao, Medicinal plants, Ethnobotany, Traditional knowledge

## Abstract

**Background:**

This study explores the medicinal plant knowledge of the Baiku Yao, a unique ethnic group in China. Despite existing research on their ethnobotanical practices, a comprehensive understanding of their medicinal flora remains lacking. This study aims to document and analyze the species distribution, utilization, and traditional knowledge of medicinal plants used by Baiku Yao.

**Methods:**

Ethnobotanical surveys were conducted in various Baiku Yao villages across different seasons from 2019 to 2023. Informants were interviewed, and plant specimens were collected and identified. Statistical analyses, including the Relative Frequency of Citation (RFC), were employed to understand plant importance in Baiku Yao culture.

**Results:**

In an ethnobotanical survey conducted in the Baiku Yao region, 434 medicinal plant species were documented, highlighting significant ethnobotanical diversity and a deep cultural integration of traditional medicinal practices. The study revealed pronounced geographical variations in plant knowledge among villages, with a notable reliance on wild plants, as 85.48% were sourced from the wild, reflecting unique local ethnobotanical knowledge. Predominantly herbs and shrubs were used due to their accessibility and abundance in the local environment. High Relative Frequency of Citation (RFC) values for certain species underscored their importance for local health needs and additional economic value. The utilization of various plant parts, particularly whole plants, roots, and leaves, indicates a holistic approach to medicinal applications, adapted to combat prevalent health issues such as skin and infectious diseases. The study also uncovered the Baiku Yao’s cultural practices for countering “Gu” afflictions—a range of pathogenic conditions—with 18 diverse antidote plants used for skin, digestive, and musculoskeletal disorders. The study underscores the imperative of preserving this rich medicinal heritage through innovative models that engage youth and leverage new media, ensuring the inheritance and evolution of Baiku Yao’s traditional knowledge.

**Conclusions:**

Baiku Yao’s medicinal plant use reflects a deep, culturally ingrained knowledge, closely tied to local ecology. The study highlights the importance of preserving this unique ethnobotanical heritage and suggests interdisciplinary approaches for future research.

**Supplementary Information:**

The online version contains supplementary material available at 10.1186/s12906-024-04545-8.

## Introduction

The history of medicinal plants can be traced back to ancient times when various civilizations and old countries extensively used local plants for healing purposes [1]. Medicinal plants not only play an essential role in traditional medicine but have also gained attention from the modern medical field in recent years [2]. The study of medicinal plants spans various areas, such as botany, chemistry, and pharmacology, offering vital insights for drug exploration and advancement [3]. Many traditional herbs have been used to develop new drugs and produce health supplements and cosmetics, enriching the resource pool of modern medicine and biotechnology [3, 4].

In China, the study of medicinal plants, crucial to both biodiversity and cultural heritage, is advancing rapidly in the field of medical ethnobotany. This area has gained attention by integrating traditional practices with modern science, leading to the identification of over 10,000 species used in traditional Chinese medicine [5]. These plants are important to both traditional healthcare and modern pharmacology [5]. However, challenges like habitat destruction and climate change have made their conservation urgent [6]. While initiatives to document, safeguard, and sustainably use these plants are in progress, they continue to face significant threats from overharvesting, the impacts of climate change, and increasing urbanization [6]. Against these backgrounds, the Baiku Yao, a distinctive subgroup within the Yao people of China, have drawn our interest because of their distinct and unrecorded traditional medicinal knowledge.

The Baiku Yao (in Chinese “白裤瑶”) is an essential branch of the Yao ethnic group in China, mainly distributed across the Guangxi Zhuang Autonomous Region and Guizhou Province in southern China [7]. The Baiku Yao, named after the distinctive white knee-length pants worn by adult males, are renowned for their unique lifestyle, cultural customs, and knowledge of medicinal plants. They have been recognized by UNESCO as one of the most intact ethnic groups to have preserved their heritage and are hailed as “living fossils of human civilization” [7]. The Baiku Yao’s daily life is closely intertwined with nature, as their habitats encompass diverse geographical environments, including mountains, hills, and valleys. This environment enables them to possess profound knowledge and utilize various plant resources. Meanwhile, the Baiku Yao uphold ancient ways of living, including traditional farming, distinctive clothing, and cultural customs, forming a unique ethnic characteristic.

Recent studies have demonstrated that the Baiku Yao possesses abundant traditional knowledge of plant utilization. For instance, Hu et al. (2022) investigated the Baiku Yao’s use of plants for conventional dyeing of costumes, revealing their ingenious traditional dyeing techniques in textile and costume culture [8]. Additionally, research by Hu et al. focused on changes in home gardening among the Baiku Yao after village relocation, providing insights into the evolution of interactions between this ethnic group and their environment [9]. Moreover, ethnobotanical surveys by Luo et al. on traditional fodder plants and veterinary plants used by the Baiku Yao unveiled their plant applications in animal husbandry [10, 11]. Recent research has also shifted focus to edible plants among the Baiku Yao. Most recently, Luo et al. examined the nutritional value and economic potential of *Lindera pulcherrima* var. *attenuata* leaves, an edible plant used by the Baiku Yao, contributing valuable knowledge about their food culture and sustainable development [12].

While previous studies have explored the Baiku Yao’s utilization of plants for food, veterinary applications, dyeing, and fodder, a significant gap persists in our understanding of their use of medicinal plants. However, Yao medicine is a crucial aspect of traditional Chinese Medicine, particularly within the domain of ethnic medical practices. The Baiku Yao, a subgroup of the Yao people, hold an exceptionally rich traditional knowledge of medicinal plants, marking a cultural treasure within ethnic traditions. Yet, this repository of traditional medicinal plant knowledge is in decline, under threat from the expansion of Western medicine, modern medical practices.

Against this backdrop, the present study aims to study and document the species, distribution, utilization, and traditional knowledge of Baiku Yao medicinal plants through ethnobotanical investigations to contribute to preserving and inheriting their therapeutic plant culture. By synthesizing previous findings and conducting more in-depth field surveys, we expect to elucidate the characteristics, values, and significance of medicinal plants in the lives of the Baiku Yao. Integrating earlier research outputs with intensive new fieldwork will hopefully reveal insights into the features, merits, and importance of Baiku Yao medicinal plants.

## Methods

### The study area

The Baiku Yao are primarily distributed across Lihu and Baxu Townships in Nandan County, Guangxi, as well as Yaoshan Township in Libo County, Guizhou [11]. Lihu Yao Township in Nandan County is located in the northeastern part of Nandan County. The area covers 361.77 square kilometers and administers two communities and twelve administrative villages; it has a registered population of over 25,000 people, predominantly of the Baiku Yao ethnicity [13]. Baxu Township, situated in the southeastern part of Nandan County, spans an area of 510.42 square kilometers, comprising three communities and fifteen administrative villages, with a registered population of over 30,000 people, mainly from the Baiku Yao [13]. Yaoshan Township in Libo County is located in the southwestern part of Libo County, with a total area of 190.48 square kilometers. It governs eight administrative villages and has an approximate registered population of 10,000 people, predominantly of the Baiku Yao ethnicity [13]. Lihu, Baxu, and Yaoshan Townships are closely connected, located at the southern edge of the Yunnan-Guizhou Plateau, serving as a transitional zone between the plateau and the hills of Guangxi Province. This region is characterized by karst peak cluster landforms, featuring more mountains than flat land. All three townships share a subtropical monsoon climate, with cold winters, warm summers, cool springs, and autumns, and a short frost period. The complex topography and suitable climate contribute to a rich diversity of plant species.

**Fig. 1:**
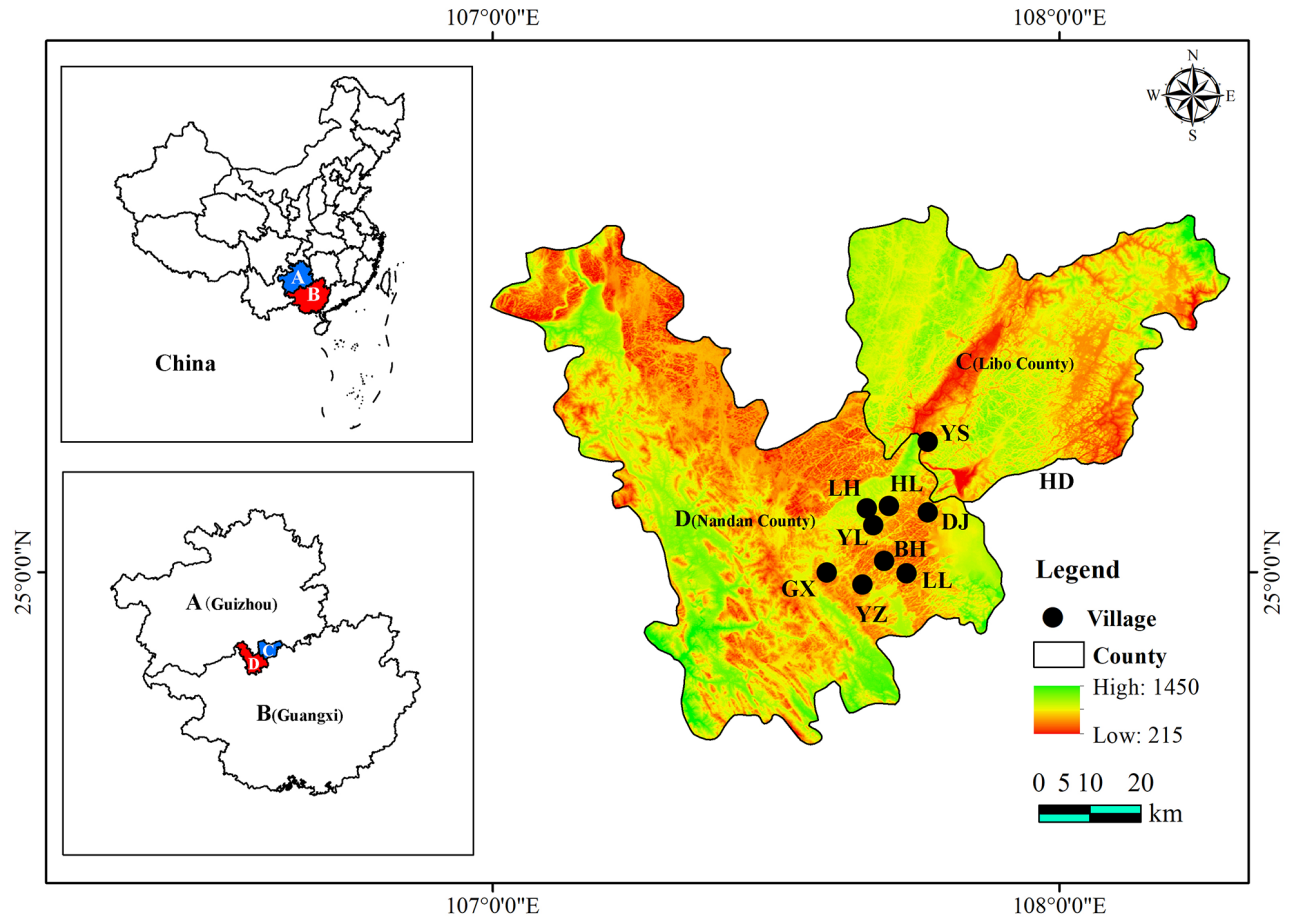
The study area (**A**: Guizhou Province; **B**: Guangxi Zhuang Autonomous Region; **C**:Libo County; **D**: Nandan County; YS: Yaoshan, HL: Huaili, LH: Lihu, YL: Yaoli, DJ: Dongjia, BH: Baha, LL: Lile, YZ: Yaozhai, GX: Guanxi.)

Based on a review of previous literature and preliminary survey findings, as well as recommendations from curators of the Baiku Yao Ethnic Museum, the following locations were selected as study sites (Fig. 1): Huaili Village, Lihu Community, and Dongjia Village in Lihu Yao Ethnic Township; Yaozhai Village, Lile Village, Baha Village, and Guanxi Village in Baxu Township, Nandan County, Guangxi; and Yaoshan Village in Yaoshan Township, Libo County, Guizhou. These sites were chosen as major settlements of the Baiku Yao, where traditional culture remains well preserved, facilitating data collection. From 2019 to 2023, we carried out a total of 8 ethnobotanical survey trips to Baiku Yao villages across all four seasons.

### Data collection

To grasp the overall use of medicinal plants among Baiku Yao residents, we employed ethnobotanical interviews in surveying villagers across the study sites [14]. A total of 98 respondents were interviewed (59 males and 39 females); among them, 46 were key interviewees. The key interviewees were primarily selected using purposive sampling from traditional healers, well-known elderly residents, village officials, and others identified in preliminary surveys (who had accumulated extensive traditional knowledge of medicinal plants used by the Baiku Yao) [15]. Additionally, 52 general respondents were chosen using the snowball sampling technique from among neighborhoods and medicinal markets. The ages of the respondents ranged from 31 to 82 years, all of whom were local residents of the Baiku Yao community. Throughout the study, informed consent was obtained from each interviewee prior to conducting interviews. The data collection on the traditional medicinal plant knowledge of the Baiku Yao primarily took place from August 2019 to May 2023. It was mainly conducted through semi-structured interviews, supplemented by participatory surveys and field investigations, to document the plant taxa, vernacular names, used parts, uses, and distribution associated with the medicinal plants used by the Baiku Yao.

This study also utilized the relative frequency of citation (RFC) for statistical analysis. RFC values were calculated using the following formula [16]:

$$RFC = \frac{{{\rm{FCs}}}}{{\rm{N}}}$$

FCs represent the total number of mentions for a given plant species across all informants, and N represents the total number of informants [16]. RFC values range from 0 to 1, with higher values indicating a closer association between the medicinal plant and the daily life of the Baiku Yao [17].

Plant specimens were collected or photographed during field observations with participants. Specimens were later identified by consulting local people or using online databases including the Flora of China (http://www.efloras.org/), Flora Republicae Popularis Sinicae (http://www.iplant.cn/frps), World Flora Online (https://www.worldfloraonline.org/), Chinese Field Herbarium (http://www.cfh.ac.cn/), Global Biodiversity Information Facility (https://www.gbif.org/), National Specimen Information Infrastructure (http://www.nsii.org.cn/2017/home.php), and Plant Photo Bank of China (http://ppbc.iplant.cn/). The Flora of China and Angiosperm Phylogeny Group IV system (APG IV) were used for scientific nomenclature. All specimens were preserved in the Herbarium of the Guangxi Zhuang Autonomous Region, Institute of Traditional Chinese Medicine (GXMI).

## Results and discussions

### Taxonomic distribution

The present survey documented 434 medicinal plant species used traditionally by the Baiku Yao. These plants belonged to 331 genera and 122 families (Appendix Table 1).

At the family level, most medicinal plants were concentrated in a few significant families (Table 1). Specifically, 33 families contained ≥ 5 medicinal species, comprising 288 species and accounting for 66.36% of the total species; 35 families contained 2–4 medicinal species, including 95 species and accounting for 21.89% of the total species; 54 families had only one medicinal species each, comprising 54 species and accounting for 12.44% of the total species.

At the genus level, medicinal plants were more evenly distributed (Table 1). Only 5 genera contained ≥ 5 medicinal species, comprising 25 species and accounting for 5.76% of the total species; 69 genera contained 2–4 medicinal species, comprising 152 species and accounting for 35.02% of the total species; most genera contained only one medicinal species each, with 257 monotypic genera comprising 257 species and accounting for 59.22% of the total species.

**Table 1:** Distribution of families and genera across species

Type	Number of Families	Number of Species	Proportion of Total Species (%)	Type	Number of Genera	Number of Species	Proportion of Total Species (%)
Multi-species Families (≥ 5 species)	33	288	66.36	Multi-species Genera (≥ 5 species)	5	25	5.76
Few-species Families (2–4 species)	35	95	21.89	Few-species Genera (2–4 species)	69	152	35.02
Single-species Families (1 species)	54	54	12.44	Single-species Genera (1 species)	257	257	59.22
**Total**	**122**	**434**	**100**	**Total**	**331**	**434**	**100**

In summary, Baiku Yao medicinal plants were primarily concentrated in a few major families, while distribution at the genus level was more dispersed, mainly comprising monotypic and oligotypic genera. The taxonomic composition may be related to local vegetation types and the distribution of plant resources. The taxonomic distribution also reflects the Baiku Yao’s preference for and widespread use of medicinal plants from certain families and genera.

### Life form and resource types

**Fig. 2:**
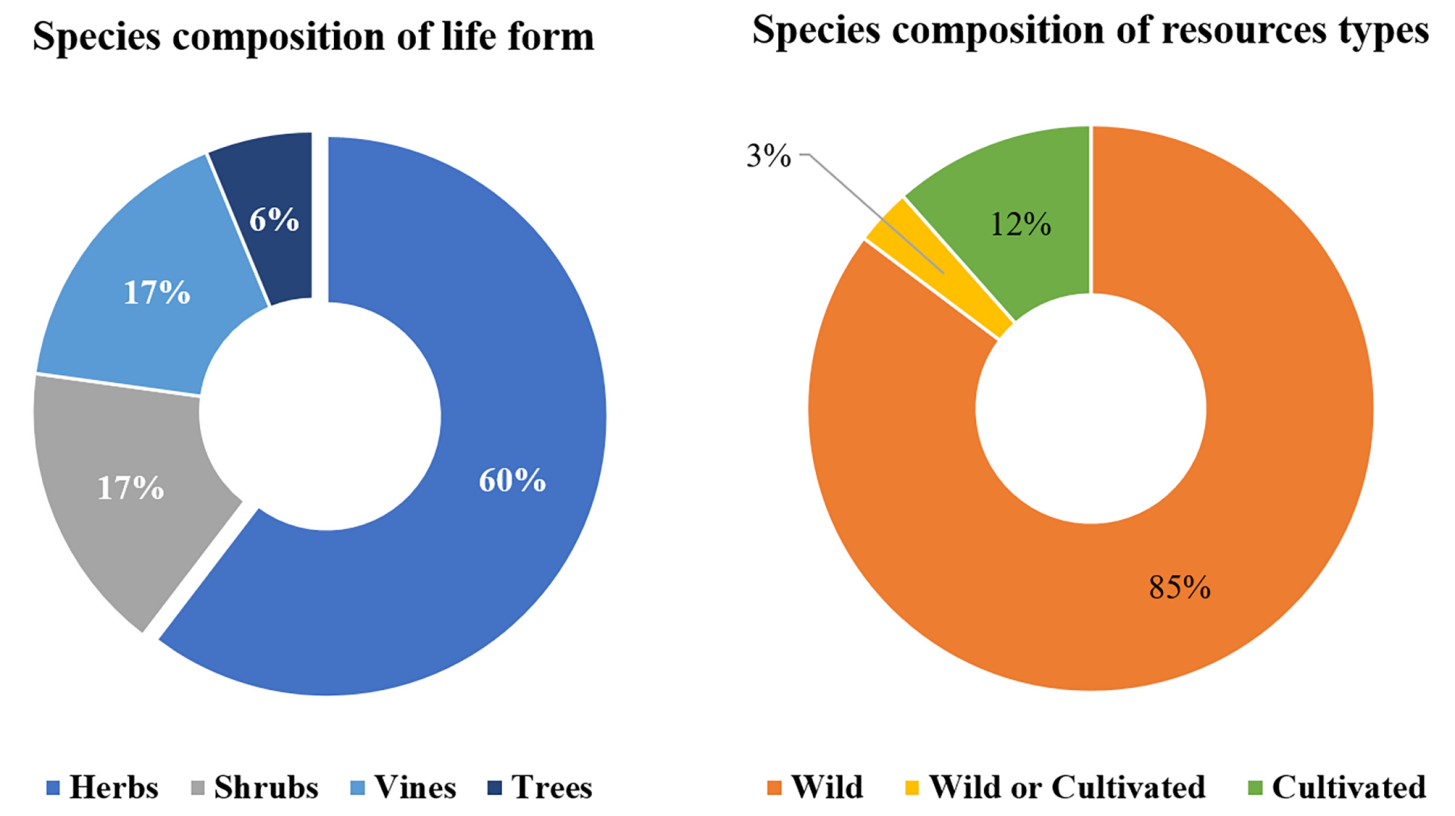
Species compositions of growth types and resources types

Regarding life forms (Fig. 2), most medicinal plants we recorded were herbs, comprising 262 species and accounting for 60.37% of the total species. The following are shrubs (73 species, 16.82%), vines (72 species, 16.59%), and trees (27 species, 6.22%). The life form composition indicates herbs and shrubs as the primary sources of Baiku Yao medicinal plants, likely related to local ecological environments and vegetation where herbs and shrubs are common. Moreover, herbs and shrubs are relatively more straightforward to collect and use, making them more prevalent in medicinal applications. Vines were also considerably used by the Baiku Yao for medicine; local healers believe vines have excellent therapeutic efficacy, especially for treating rheumatism, promoting blood circulation, and removing blood stasis.

Regarding resource types, as it is shown in Fig. 2, most medicinal plants (371 species, 85.48% of the total) were wild, while a small portion (63 species, 14.52%) were cultivated. The recorded resource types signify a significant reliance on wild resources, potentially attributable to local biodiversity and the Baiku Yao’s extensive wild-harvesting experience. The dependence on wild medicinal plants also highlights the uniqueness of Baiku Yao’s ethnobotanical knowledge.

Baiku Yao medicinal plants were primarily wild herbs and shrubs commonly found locally, with wild sources dominant, which aligns well with local ecological characteristics. The high reliance also reflects the Baiku Yao’s rich traditional knowledge of medicinal plants.

During in the survey in the Baiku Yao region, it was documented that 11 plant species are designated as second-class nationally protected plants in China. These species are: *Dysosma versipellis*, *Bletilla striata*, *Angiopteris fokiensis*, *Fagopyrum dibotrys*, *Paris cronquistii*, *Paris polyphylla*, *Paris fargesii*, *Corydalis saxicola*, *Alsophila spinulosa*, *Paphiopedilum micranthum*, *Sophora tonkinensis*. While certain species within the genus *Paris* can be substituted with cultivated varieties, which are already widely propagated, nearly all other species lack alternatives. The protection of these plants is essential for the maintenance of biodiversity. Concurrent utilization and conservation require the establishment of stricter collection restrictions, the promotion of artificial cultivation to lessen reliance on wild populations, and the development of alternative economic activities to reduce dependence on these medicinal plants. Such measures are imperative to ensure both ecological and economic sustainability, thereby facilitating the preservation of natural resources and fostering long-term prosperity within local communities.

### Medicinal part used

**Fig. 3:**
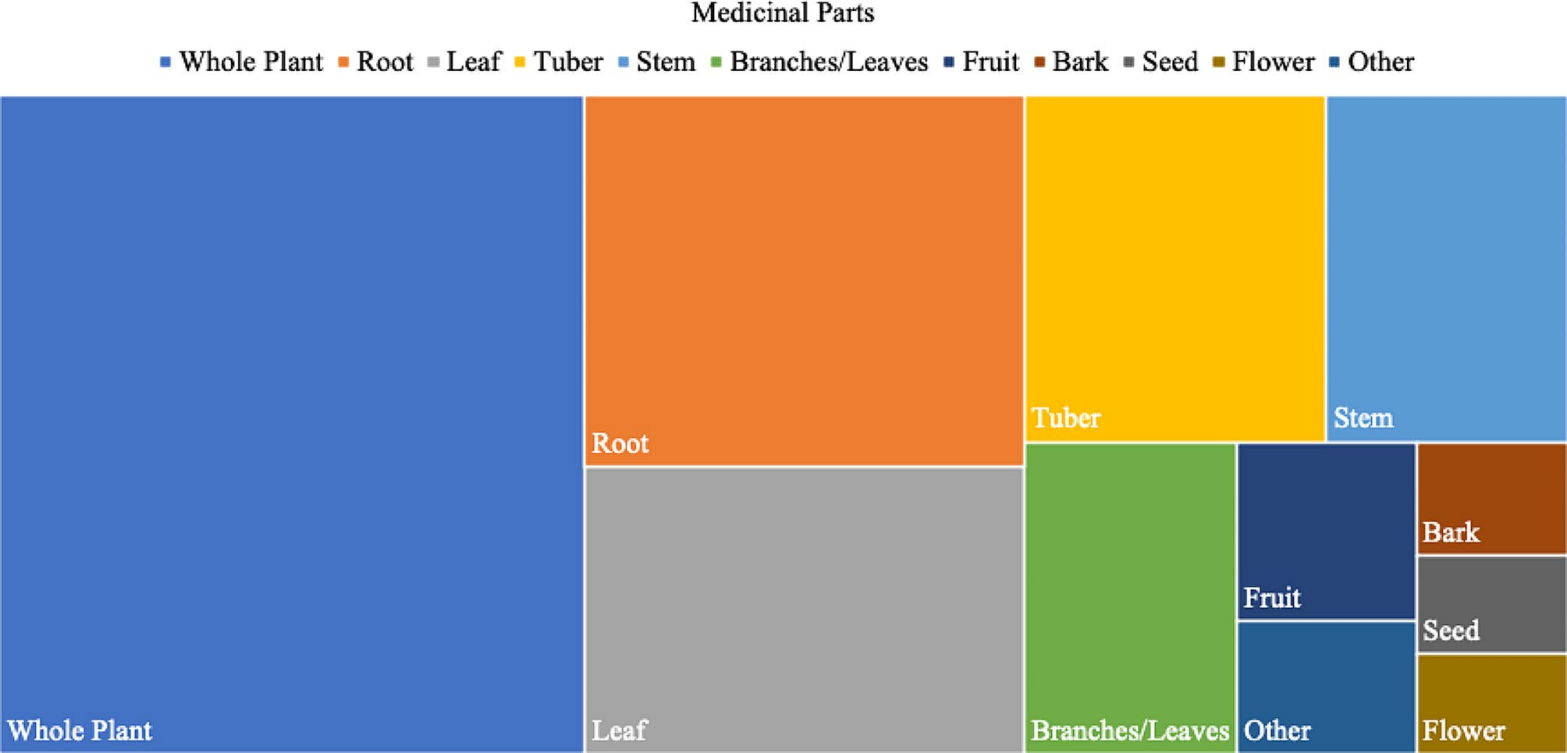
Medicinal parts of plants used in the Baiku Yao community

Statistical results showed that Baiku Yao medicinal plants involved 11 major plant parts (Fig. 3). Whole plant was the most common (180 species, 37.34% of total), followed by root (76 species, 15.77%), leaf (mainly tender leaf, 59 species, 12.24%), and rhizome (including tuber and bulb, 49 species, 10.17%).

Beyond the most frequently used parts above, Baiku Yao medicinal plants also involve stem (including rhizome and old stem), branch and leaf, fruit, seed, flower, etc. The medicinal parts recorded reflect the Baiku Yao’s holistic knowledge and use of various plant structures.

The prevalence of whole-plant use may be attributable to Baiku Yao’s belief that medicinal constituents are evenly distributed in many herbs. Thus, the entire plant can be fully extracted and utilized for therapeutic efficacy. Meanwhile, roots and leaves are essential storage organs and photosynthetic organs of many plants, also rich in medicinal constituents, making them valued medicinal parts.

Baiku Yao medicinal plants involve diverse and versatile use of multiple parts, emphasizing both specific part values and the synergistic effects of whole plants. This reflects Baiku Yao’s sophisticated ability to identify the pharmacological properties of plant structures and their holistic utilization of medicinal plants.

### Processing and targeted illness

This study conducted a statistical analysis of the Baiku Yao’s medicinal plant processing methods and the illnesses commonly addressed by these practices. We documented eight processing methods and fifteen disease categories, detailed in Tables 2 and 3.

The analysis of processing methods revealed that “crushing for external use” was the most common, accounting for 36.39% of all mentions. This prevalence suggests a cultural preference for topical applications, which may offer immediate and localized relief for conditions such as traumatic injuries, burns, and snake bites. The methods of “decoction for oral use” and “soaking in alcohol for oral use” were also frequently mentioned, constituting 24.31% and 11.25% of the records, respectively. These methods are essential for extracting medicinal properties that are water or alcohol-soluble and are effective for systemic ailments like respiratory diseases and digestive system disorders.

**Table 2:** The processing methods of medicinal plants by Baiku Yao

Processing Method	Frequency of Occurrence	Percentage of Total Frequency (%)
Crush for external use	262	36.39
Decoction for oral administration	175	24.31
Soak in alcohol for oral administration	81	11.25
Boil for external washing	75	10.42
Medicinal diet	51	7.08
Eat directly	28	3.89
Soak in alcohol for external use	19	2.64
Others	29	4.03

The analysis of Baiku Yao medicinal plant use, as detailed in Table 3, highlights the community’s primary health concerns. Diseases of the digestive system were the most frequently mentioned, constituting 18.42% of total mentions. This prevalence is consistent with the dietary habits and environmental conditions of the Baiku Yao, suggesting that gastrointestinal issues are a significant health concern, possibly exacerbated by the local climate and agricultural lifestyle, which can influence diet and gut health.

Traumatic injuries, including sprains and musculoskeletal system diseases, were the second most cited category, making up 15.46% of mentions. The prominence of such conditions reflects the physical nature of the Baiku Yao’s daily activities, with labor-intensive farming practices leading to a higher incidence of falls and related injuries.

Skin diseases, accounting for 13.04% of the mentions, are likely influenced by the local humid and hot climate, which can predispose individuals to a variety of dermatological issues. The respiratory system was another common concern, with 9.68% of mentions, which may be related to both environmental factors and indoor air quality, especially in a community engaged in agriculture where exposure to dust and organic matter is common.

**Table 3:** The targeted illness of medicinal plants by Baiku Yao

Category	Frequency Mentioned	Percentage (%)
Digestive System	236	18.42
Traumatic Injury and Sprain (Musculoskeletal System Diseases)	198	15.46
Skin Diseases	167	13.04
Respiratory System	124	9.68
Poisonous Insect Bites and Poisoning	87	6.79
Gynecological Problems	75	5.85
Urinary System	75	5.85
Nerves and Psychosomatic Problems	63	4.92
Circulatory System	63	4.92
Strong Body and Relieve Pain	54	4.22
ENT (Ear, Nose, Throat) or Ophthalmology	41	3.2
Other Uses	40	3.12
Immune System	34	2.65
Heat Clearing and Detoxifying	24	1.87

In summary, Baiku Yao’s medicinal processing and applications are highly correlated with prevalent local illnesses. External preparations are primarily used for injuries, while internal practices treat systemic diseases. The same plant can also be processed differently to treat various conditions. The medicinal plants we recorded fully demonstrate the abundant, targeted, and practical nature of Baiku Yao ethnobotanical knowledge. Such a unique therapeutic culture is worth further research and protection.

Additionally, understanding the Baiku Yao’s geographical setting and way of life enriches the context of their medicinal practices. Inhabiting humid tropical and subtropical highlands and primarily engaged in corn and rice cultivation, the Baiku Yao community is prone to occupational musculoskeletal injuries. The local climate also contributes to the prevalence of skin and infectious diseases. The selection and application of medicinal plants by the Baiku Yao are evidently tailored to their specific environmental conditions and health challenges, reflecting a sophisticated adaptation to their natural and societal environment.

### Quantitative analysis

RFC values reflect the close association between plants and local ethnic groups’ lives. Plants with higher RFC values see more extensive and frequent application in Baiku Yao daily life. Notably, only plants with RFC ≥ 0.4 are listed in the Table 4, while in fact, Baiku Yao medicinal plant use has enormous diversity, with most plants having lower RFCs. The RFC value of different species signifies the remarkable diversity of Baiku Yao’s ethnobotanical knowledge.

**Table 4:** the medicinal plants recorded with RFC ≥ 0.4

No.	Species Name	RFC
1	*Eriobotrya japonica* (Thunberg) Lindley	0.537
2	*Rosa laevigata* Michaux	0.537
3	*Agrimonia pilosa* Ledebour	0.4722
4	*Disporopsis pernyi* (Hua) Diels	0.4722
5	*Gynostemma pentaphyllum* (Thunberg) Makino	0.463
6	*Nephrolepis cordifolia* (L.) C. Presl	0.463
7	*Eucommia ulmoides* Oliver	0.4537
8	*Achyranthes bidentata* Blume	0.4444
9	*Artemisia argyi* H. Leveille & Vaniot	0.4444
10	*Curculigo orchioides* Gaertner	0.4444
11	*Toddalia asiatica* (Linnaeus) Lamarck	0.4444
12	*Zanthoxylum armatum* Candolle	0.4444
13	*Mucuna birdwoodiana* Tutcher	0.4259
14	*Sophora tonkinensis* Gagnepain	0.4259
15	*Clerodendrum bungei* Steudel	0.4167
16	*Rohdea japonica* (Thunberg) Roth	0.4167

Statistical data showed the highest RFC values for *Eriobotrya japonica* and *Rosa laevigata*, likely due to their multifunctional edible and medicinal value, which is very familiar to the locals. Analysis of high RFC species revealed the widespread use of medicinal food plants in Baiku Yao daily life, such as *Rosa laevigata*, *Eucommia ulmoides*, *Achyranthes longifolia*, *Dioscorea persimilis*, and *Phytolacca americana*. The Baiku Yao region has long suffered from barren lands and scarce resources, making nutritional deficiencies common and driving unique preferences for invigorating plants. Additionally, many high RFC species, like *Agrimonia pilosa*, *Disporopsis pernyi* and *Gynostemma pentaphyllum*, possess hemostatic properties. For instance, modern research has validated anti-inflammatory compounds in *Agrimonia pilosa* that facilitate wound healing, consistent with its traditional use by the Baiku Yao for hemostasis [18]. This pharmacological feature adapts to the higher incidence of trauma in the Baiku Yao’s native humid and hot karst terrain. Similar patterns occur in ethnobotanical uses by geographically proximate groups like the Mulam people [19]. Interestingly, though not mentioned in every village, most anti-witchcraft (Gu poisons) plants had relatively high RFCs. During our survey, we noticed such plants being grown and recognized by most households in the few villages mentioning them.

Based on the above analysis, plants with high RFCs generally have three key traits: (1) additional economic values beyond medicinal uses; (2) main medicinal effects suiting local needs, e.g., trauma and hemostasis; (3) abundant local populations and resources. These frequently used plants are integral to Baiku Yao ethnobotanical culture, with traditional knowledge and use warranting emphasis on preservation and inheritance.

### Differences among villages

**Fig. 4:**
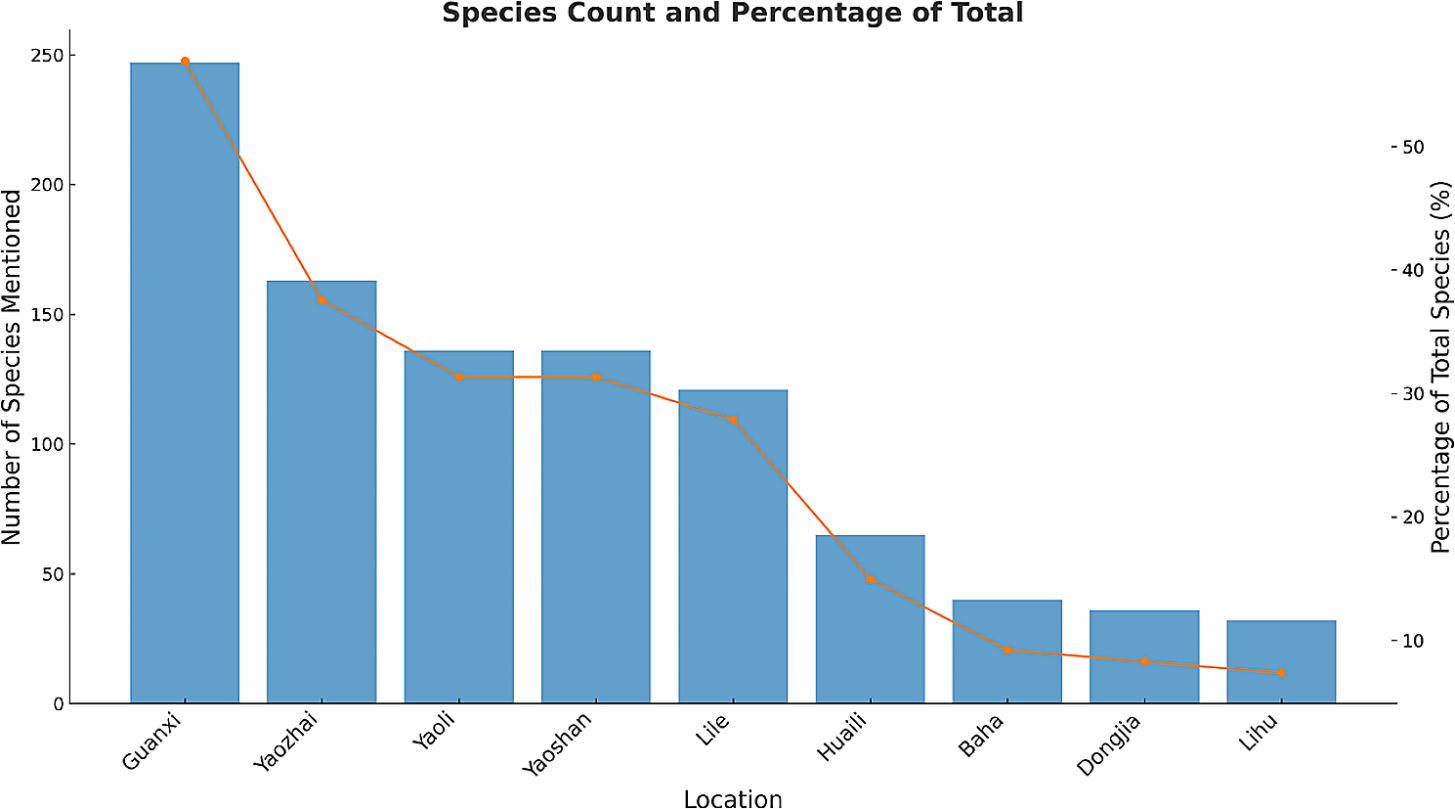
Species count and percentage in different villages

This study investigated ethnobotanical knowledge across 9 Baiku Yao villages, documenting 434 medicinal plant species. Results found pronounced geographical variations in medicinal plant knowledge and use between villages of the same ethnic group (Fig. 4). For instance, our survey documented 247 species in Guanxi Village, the largest sample, accounting for 56.91% of the total recorded species. Dongjia Village, where we collected the smallest sample capacity, only reported 36 species, a mere 8.29% of the total. This discrepancy is mainly attributable to Guanxi Village having more experienced traditional healers and richer inherited ethnobotanical knowledge, while Dongjia Village no longer has traditional healers. Comparative analysis across villages reflects distinct geographical patterns in ethnobotanical knowledge. Most medicinal plants were only mentioned in 1–2 villages without widespread circulation. Two key factors shape this distribution: First, transportation inconveniences and cultural integration result in knowledge being largely confined within villages, limiting diffusion. Second, medicinal plant gathering in each village mainly occurs in surrounding areas to be self-sufficient, restricted by local vegetation. Thus, local knowledge is heavily influenced and constrained by proximate flora. Additionally, the presence of traditional healers directly impacts knowledge richness, as they are both inheritors and innovators advancing its inheritance.

This study’s distribution patterns reflect the region’s abundant yet fragmented medicinal plant knowledge. The distribution relates closely to geographical isolation and villages’ self-sufficient lifestyles. Findings reveal unique local cultural features and underscore preserving this traditional medicinal culture. Enhanced exchange between villages, regional medicinal plant gardens, ethnobotanical compendiums, and scientific research are recommended to better safeguard local ethnobotanical heritage.

### Antidote for Gu poisons

In Southeast Asia and among the ethnic regions in southern China, the term “Gu” (in Chinese called “蛊”) carries a mystique [20, 21]. Numerous ancient Chinese medical texts contain various interpretations of Gu: firstly, it is described as venomous creatures from the natural world or those artificially cultivated; secondly, it is perceived as a form of poison or a manifestation of intense pathogenic heat in the natural world; others consider Gu as an early understanding of bacteria by ancient people; and some view Gu as a term denoting a disease or a pathological condition [21, 22]. In summary, traditional Chinese medicine offers a diverse range of interpretations for Gu, and as of now, a unified definition remains elusive [23].

According to our interview with the local community, Baiku Yao believes that Gu represents an insect specially bred by Gu practitioners. These insects are nourished with the blood and flesh of the practitioner and are periodically transferred to other individuals. According to the local folklore, if the Gu practitioner does not transmit the Gu to another person, they will suffer the consequences of the Gu turning on them, potentially leading to their demise.

In their prolonged struggle against “Gu,” the Baiku Yao people have developed a distinct set of diagnostic and therapeutic theories and methods. Baiku Yao acknowledges the existence of various types of “Gu,” with common examples including Ant Gu, Frog Gu, and Bone Gu. Symptoms such as unexplained abdominal pain, sensations of throat obstruction, and breathing difficulties are associated with Frog Gu; the perception of foreign objects moving within the skin and tissue indicates Ant Gu, whereas pain within the bones, often with shifting locations, is indicative of Bone Gu. A key feature of Gu affliction is the constant migration of pain, making it challenging to pinpoint the specific cause.

To prevent and manage Gu afflictions, Baiku Yao has devised unique strategies. They strongly avoid individuals known to practice Gu, refraining from intermarriage or cohabitation with them, minimizing contact, and isolating those involved in Gu practices to cut off the source of Gu. Furthermore, as a precaution, each household cultivates several antidotes for Gu in their homegardens. Additionally, the local community preserves and disseminates knowledge about antidotes for Gu and traditional practices, effectively preventing the occurrence of Gu poison.

Interestingly, our survey found that antidotes for Gu afflictions are not uniformly distributed across all Baiku Yao villages, and their prevalence varies significantly. Overall, the closer a village is to well-developed transportation networks, the higher the diversity of Gu antidotes and the frequency of their mention. In contrast, more remote villages tend to have fewer antidotes. For instance, in the Yao village that once served as the township government center, we documented as many as 10 different Gu antidote plants. In contrast, in the more isolated Dongjia Village, only 2 were recorded. Our interviews show that transient populations from outside have introduced Gu, which is not an indigenous tradition. Consequently, Gu afflictions are more common in villages with better transportation access, and local residents have a greater understanding of Gu antidotes. However, these communities have continuously developed skills and methods specific to their culture through their prolonged struggle against diseases.

**Fig. 5:**
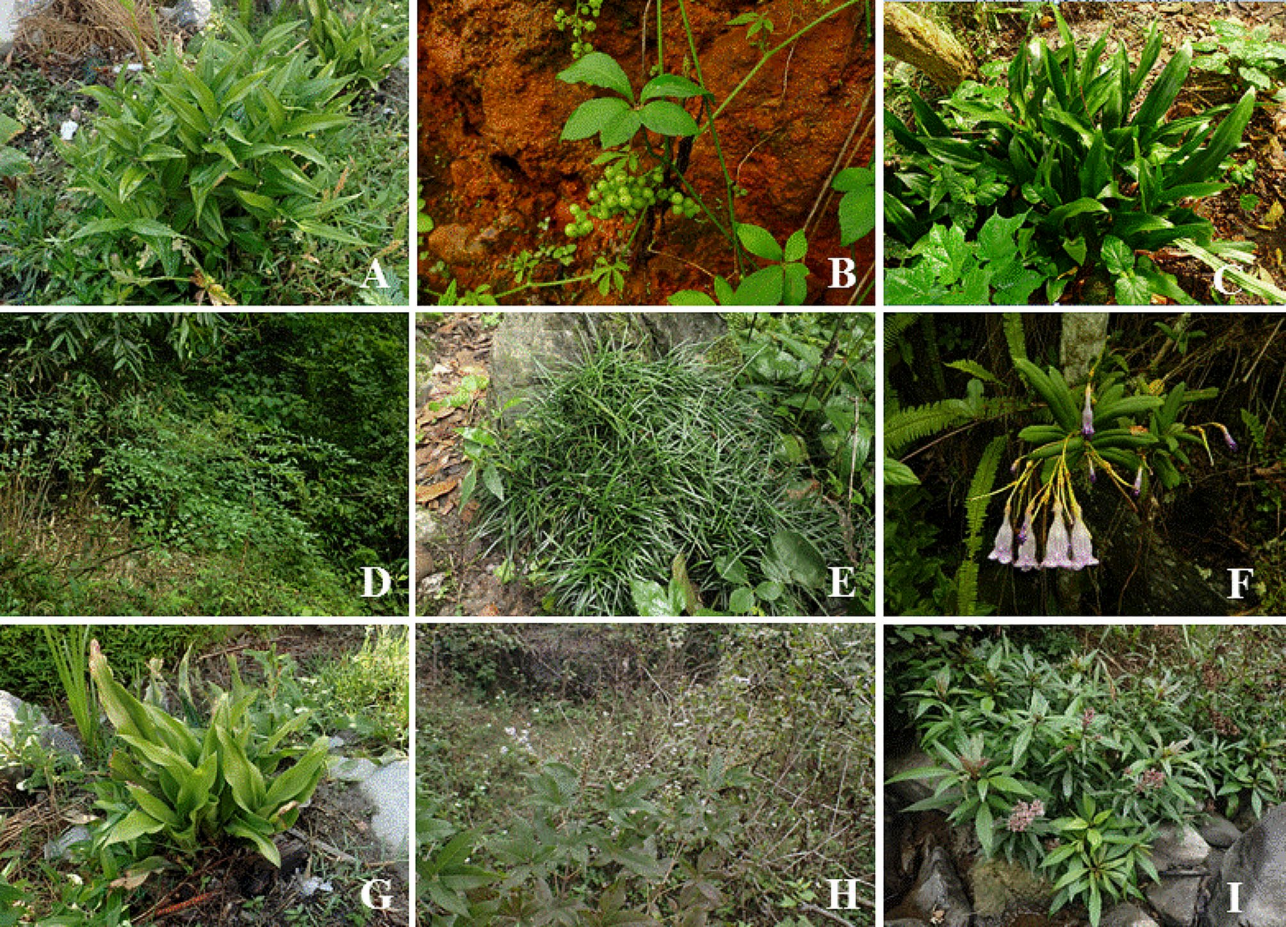
partial medicinal plants used by the Baiku Yao for counteracting “Gu” poison (**A**: *Disporopsis pernyi*, **B**: *Gynostemma pentaphyllum*, **C**: *Rohdea japonica*, **D**: *Nandina domestica*, **E**: *Liriope spicata*, **F**: *Lysionotus pauciflorus*, **G**: *Crinum asiaticum* var. *sinicum*,**H**: *Vitex negundo*, **I**: *Eupatorium fortunei*.)

In our investigation, we identified a total of 18 different Baiku Yao Gu antidote plants belonging to 14 families and 17 genera (Fig. 5), demonstrating that Baiku Yao Gu antidotes are taxonomically diverse and not concentrated within specific families or genera. When categorizing these Gu antidote plants by their secondary therapeutic uses, we found that all of them are employed to treat various other ailments locally. Among these, 14 are primarily used to treat injuries, promote blood circulation, and resolve blood stasis, while 7 are used for gastrointestinal disorders (such as stomach pain, diarrhea, and abdominal discomfort), 5 for skin conditions (including itching, athlete’s foot, and psoriasis), and 5 for treating snakebites, poisoning, and rabid dog bites. Comparing these remedies with the three most common types of Gu afflictions (Ant Gu, Frog Gu, and Bone Gu), it becomes evident that Gu afflictions often involve skin, digestive system disorders, and the musculoskeletal and nervous systems. Correspondingly, many of the Gu antidotes are frequently used to treat these specific categories of ailments, suggesting that the selection of medicinal plants by the Baiku Yao people for treating Gu afflictions has a rational basis. Throughout their extensive experience and accumulation of knowledge in combating Gu poison over the years, they have developed rich traditional expertise, which warrants further in-depth research.

### Inheritance challenges

As a unique cultural heritage of the Baiku Yao, the inheritance of ethnobotanical knowledge currently faces multiple threats and challenges. Firstly, changes in transmission methods. Traditionally, knowledge was passed down orally from healers to apprentices. However, this singular master-disciple model is increasingly unsustainable due to societal shifts. On the one hand, young migrant workers have limited time back home for systematic learning. On the other, declining marriage rates also impede intergenerational transmission. Secondly, lifestyle changes. Due to socioeconomic development, poverty alleviation policies, and more, Baiku Yao society is changing tremendously from subsistence farming to migrant labor. As children and youth gradually detach from traditional agriculture, their ability to identify surrounding plants weakens. Urbanization attracts youth to leave villages, distancing them from ethnobotanical culture. Finally, modern lifestyles cannot be ignored. Exposure to external cultures and reliance on pharmaceuticals has reduced the perceived value of traditional medicine among youth, weakening motivations for knowledge transmission.

Despite multifaceted pressures, some youths are proactively safeguarding this heritage. A few tech-savvy young Yao creatively leverage platforms like TikTok and Kuaishou to disseminate ethnobotanical knowledge. This positive phenomenon aligns with contemporary youths’ information consumption habits, expanding the reach of medicinal culture and public awareness. By transforming knowledge into economic value, medicinal plants provide youth livelihoods and motivations for sustained usage, enabling effective integration of cultural traditions and modern communication. Such inheritance innovation models strategically utilize new media and youth creativity, providing valuable experiences for incentivizing and protecting ethnobotanical cultures worthy of promotion.

The Baiku Yao case demonstrates hope remains for knowledge inheritance given appropriate methods to motivate youth. Support and opportunities from all societal sectors are needed to encourage youth to inherit and innovate. Adapting promotional approaches to suit youth characteristics can rejuvenate medicinal culture amidst changing times. Relevant departments should also provide policy support to advance preservation jointly.

### Uniqueness of Baiku Yao medicinal plants

To explore the distinctive characteristics of medicinal plants in the Baiku Yao community, we conducted a comparative analysis with ethnobotanical studies from geographically adjacent communities of the Shui, Mulam, and Maonan peoples [24–26]. Our study documented 434 medicinal plant species within the Baiku Yao community. In comparison, the Mulam people utilized 456 species, the Shui community recorded 505 species, and the Maonan documented 368 species [24–26]. This data demonstrates that the Baiku Yao and their neighboring ethnic groups all possess a high diversity of medicinal plants, reflecting a profound understanding and utilization of local plant diversity, which, in turn, highlights the rich plant biodiversity of the region.

Regarding the scope of therapeutic applications, the Baiku Yao community specifically emphasized the use of medicinal plants in treating diseases such as skin conditions and infectious diseases. In contrast, medicinal plants among the Mulam are primarily used for digestive system diseases, while the Shui community focuses on plants for fractures and rheumatism. These differences reflect the main health challenges, lifestyle habits, and the ways in which each community utilizes local environmental resources. Our survey in the Baiku Yao community also noted applications for treating “Gu poison,” a topic not specifically addressed in the studies of the Mulam, Shui, or Maonan communities.

Moreover, our research revealed the uniqueness of the Baiku Yao community in the inheritance and protection of medicinal plant knowledge. Especially noteworthy is the exploration of using modern technological means, such as new media platforms (short video apps, etc.), to disseminate and protect knowledge of medicinal plants, a trend not reported in the literature of other communities. This phenomenon, likely due to the emerging trend of using new media to spread traditional knowledge in recent years, not only demonstrates the Baiku Yao community’s modern attempts to protect traditional knowledge but also offers new pathways for other communities to protect and inherit traditional medical knowledge.

Through comparative analysis with other ethnic communities, our study not only fills the research gap concerning the medicinal plant knowledge of the Baiku Yao community but also highlights its uniqueness and novelty in terms of medicinal plant diversity, therapeutic application range, and the protection and inheritance of traditional knowledge. These findings hold significant scientific value and societal importance for promoting the protection and inheritance of medicinal plant knowledge among ethnic minorities and its application in modern society.

## Conclusion

Through systematic ethnobotanical investigations, this study thoroughly documented and analyzed Baiku Yao medicinal plant species, used parts, processing methods, and therapeutic knowledge systems. Results show 434 documented species primarily concentrated in a few major families and genera. Local wild herbs and their tender leaves and roots are primary resources. Comprehensive utilization occurs across all plant parts, especially whole plants and roots. Diverse processing techniques and targeted therapies demonstrate high adaptation to local health issues. Many frequently used medicinal plants possess unique local pharmacology validated by science. Pronounced geographical variations exist in Baiku Yao ethnobotanical knowledge across different villages. While inheritance faces multiple challenges, innovative transmission approaches provide hope.

In this study, we have unveiled the rich ethnobotanical knowledge of the Baiku Yao people, highlighting the significance of their medicinal plant use not only for traditional healthcare but also for its potential contributions to modern medicine and conservation efforts. Our findings underscore the critical importance of preserving this knowledge for future generations, offering a gateway to understanding the interplay between humans and their environment. Looking forward, we advocate for increased interdisciplinary research to explore the phytochemical properties of these plants, develop sustainable harvesting methods, and integrate traditional wisdom into global health practices. This research not only enriches the field of ethnobotany but also opens avenues for future investigations into the conservation of medicinal plant species and the cultural heritage of the Baiku Yao, promising to bridge traditional knowledge and modern science for the betterment of both ecological conservation and human health.

### Electronic supplementary material

Below is the link to the electronic supplementary material.

Supplementary Material 1

## Data Availability

The authors confirm that the data supporting the findings of this study are available within the article [and/or] its supplementary materials.

## Abbreviations

UNESCO: United Nations Educational, Scientific and Cultural Organization
