# Supplementary material for: Medicinal flora of the baiku yao people — An ethnobotanical documentation in South China
Source: BMC Complement Med Ther. 2024 Jun 21;24:242. doi: 10.1186/s12906-024-04545-8 (PMC11193194; doi:10.1186/s12906-024-04545-8)
Supplement: Supplementary file 1 — Supplementary Material 1 [file 12906_2024_4545_MOESM1_ESM.doc]

**Appendix Table 1 Inventory of Baiku Yao Meidicnal Plants**

| **Scientific name** | **Local game** | **Family** | **Growth types** | **Resource typess** | **Used parts** | **Use methods** | **Targeted illness** | **Distributions** | **RFC** | **Voucher number** |
| --- | --- | --- | --- | --- | --- | --- | --- | --- | --- | --- |
| *Abelmoschus manihot* (Linnaeus) Medikus |  | Malvaceae | herb | wild | root | crush and apply topically | mastitis, anti-inflammatory, cancer | Guanxi, Yaozhai, Lile | 0.1204 | HRC396 |
| *Abelmoschus moschatus* Medikus |  | Malvaceae | shrub | wild | root | crush and apply topically | breast pain | Yaoli, Guanxi | 0.0741 | HRC315 |
| *Acalypha australis* Linnaeus | e nian rao | Euphorbiaceae | herb | wild | whole plant | decoct in water and take orally | diarrhea, dysentery, pediatric indigestion, nosebleed, eczema | Yaozhai, Yaoshan | 0.1574 | HRC1227 |
|  |  |  |  |  |  | crush and apply topically | traumatic injury |  |  |  |
| *Achillea millefolium* Linnaeus | e qiu | Asteraceae | herb | wild | whole plant | soak in wine and apply externally | rheumatic bone pain, traumatic swelling and pain | Yaoshan | 0.0278 | HRC1446 |
|  |  |  |  |  |  | decoct in water and take orally | toothache, acute mastitis |  |  |  |
| *Achyranthes aspera* Linnaeus | e gui nu you | Amaranthaceae | herb | wild | whole plant | decoct in water and take orally | joint loosening, fever, cough | Yaoli, Guanxi, Yaozhai, Lile, Lihu, Yaoshan | 0.3611 | HRC202 |
|  |  |  |  |  |  | soak in wine and take internally | joint pain |  |  |  |
|  |  |  |  |  |  | decoct in water and wash externally | bone setting |  |  |  |
| *Achyranthes longifolia* (Makino) Makino |  | Amaranthaceae | herb | wild | root | crush and apply topically | bone setting | Yaozhai, Lile, Yaoshan, Guanxi, Huaili | 0.4444 | HRC20 |
|  |  |  |  |  |  | stew with meat | immune boosting, menstrual disorders, prolonged menstruation |  |  |  |
| *Acmella paniculata* (Wallich ex Candolle) R. K. Jansen | zu mang mei | Asteraceae | herb | wild | flower | chew and apply topically | gum swelling and pain, toothache | Guanxi, Yaozhai | 0.0926 | HRC311 |
| *Aconitum carmichaelii* Debeaux | jiu nai hua | Ranunculaceae | herb | cultivated | tuber | soak in wine and apply externally | traumatic injury | Guanxi, Yaoshan, Yaozhai | 0.1204 | HRC632 |
|  |  |  |  |  |  | crush and apply topically | unidentified swelling and infection |  |  |  |
|  |  |  |  |  |  | soak in salt water and take internally | insecticide |  |  |  |
| *Acorus gramineus* Soland. | xi hang | Acoraceae | herb | wild | whole plant | decoct in water and wash externally | fever reduction, cold, headache | Yaozhai, Guanxi | 0.1481 | HRC656 |
| *Acorus tatarinowii* Schott |  | Acoraceae | herb | cultivated | whole plant | decoct in water and take orally | malaria, high fever | Yaozhai, Yaoshan | 0.1667 | HRC244 |
|  |  |  |  |  |  | crush and apply topically | snake bite, detoxification |  |  |  |
| *Adenostemma lavenia* (Linnaeus) Kuntze |  | Asteraceae | herb | wild | branches and leaves | crush and apply topically / decoct in water and wash externally | pus formation, child oral ulcers, herpes | Huaili, Yaozhai, Guanxi | 0.1389 | HRC608 |
| *Adiantum capillus-veneris* L. | a hao ka niao pai | Pteridaceae | herb | wild | whole plant | soak in wine and take internally | traumatic injury | Yaoshan | 0.0556 | HRC1185 |
|  |  |  |  |  |  | decoct in water and take orally | urinary retention, back pain, nocturnal emission, postpartum low milk supply |  |  |  |
| *Ageratum conyzoides* Linnaeus |  | Asteraceae | herb | wild | whole plant | Sun-dry, grind and apply externally | appendicitis, snake bite | Yaoli | 0.0278 | HRC205 |
| *Agrimonia pilosa* Ledebour |  | Rosaceae | herb | wild | whole plant | decoct in water and take orally | joint loosening, urinary tract infection, kidney stones, traumatic injury | Yaoli, Guanxi, Yaozhai, Lile, Lihu, Yaoshan, Dongjia | 0.4722 | HRC203 |
|  |  |  |  |  |  | incinerate into ashes for ophthalmic application | eye pain, blurry vision |  |  |  |
|  |  |  |  |  |  | crush and apply topically | swelling reduction |  |  |  |
|  |  |  |  |  |  | sun-dry and pulverize for topical use | bone setting |  |  |  |
| *Ajuga decumbens* Thunberg | qi lin cao | Lamiaceae | herb | wild | whole plant | soak in wine and take internally | nephritis, red and swollen eyes, pneumonia cough | Yaoli, Yaozhai, Guanxi | 0.0741 | HRC190 |
|  |  |  |  |  |  | crush and apply topically | lumbar sprain |  |  |  |
|  |  |  |  |  |  | decoct in water and take orally | hemorrhoids bleeding |  |  |  |
| *Akebia trifoliata* (Thunberg) Koidzumi | san ye lan | Lardizabalaceae | liana | wild | whole plant | soak in wine and take internally / soak in wine and wash externally | hemiplegia | Yaoli, Guanxi | 0.1481 | HRC186 |
| *Alangium chinense* (Loureiro) Harms | tong e niao | Cornaceae | shrub | wild | leaf | crush and apply topically | snake bite, corns, calluses | Yaoli, Lile, Yaozhai, Yaoshan | 0.2407 | HRC900 |
|  |  |  |  |  |  | soak in wine and take internally / decoct in water and wash externally | internal rheumatism |  |  |  |
|  |  |  |  |  |  | steam with meat | physical weakness, pediatric seizures |  |  |  |
| *Allium macrostemon* Bunge | e ma ge er | Amaryllidaceae | herb | wild | whole plant | decoct in water and take orally | appetite stimulant, stomach bloating and pain, chronic cough | Yaoshan | 0.0463 | HRC1464 |
| *Alocasia cucullata* (Lour.) Schott |  | Araceae | herb | wild | tuber | roast over fire and apply topically | toothache, unidentified swelling and infection, snake bite | Huaili, Yaozhai, Guanxi, Lihu | 0.3333 | HRC640 |
|  |  |  |  |  |  | decoct in water and wash externally | fever |  |  |  |
| *Alocasia odora* (Roxburgh) K. Koch |  | Araceae | herb | wild | tuber | crush and apply topically | snake bite (within 5 minutes of being bitten) | Yaoli, Huaili, Yaozhai | 0.1944 | HRC139 |
|  |  |  |  |  |  | heat and apply externally | unidentified swelling and infection |  |  |  |
| *Alsophila spinulosa* (Wall. ex Hook.) Tryon | long gu feng | Cyatheaceae | shrub | wild | stem | decoct in water and take orally / soak in wine and take internally | back pain, joint pain | Guanxi | 0.0741 | HRC269 |
|  |  |  |  |  |  | cut and apply externally | traumatic injury |  |  |  |
| *Amaranthus tricolor* Linnaeus |  | Amaranthaceae | herb | cultivated | leaf | take orally directly | constipation | Lile | 0.0463 | HRC501 |
| *Amorphophallus konjac* K. Koch |  | Araceae | herb | wild | tuber | crush and apply topically | snake bite (within 5 minutes of being bitten) | Lile | 0.0556 | HRC140 |
| *Ampelopsis glandulosa* (Wallich) Momiyama | bi ka | Vitaceae | liana | wild | leaf | Sun-dry, powder, and apply on affected area | external injury bleeding, scald | Yaoshan, Huaili, Yaoli | 0.1667 | HRC1229 |
|  |  |  |  |  | root bark | crush, mix with alcohol, and apply externally | fracture |  |  |  |
|  |  |  |  |  | root, stem | soak in wine and take internally / decoct in water and take orally | rheumatic pain, painful urination |  |  |  |
| *Amygdalus persica* Linnaeus |  | Rosaceae | tree | cultivated | seed（kernel） | crush and apply topically | snake bite | Lile | 0.0278 | HRC240 |
| *Anemone hupehensis* (Lemoine) Lemoine |  | Ranunculaceae | herb | wild | root | decoct in water and take orally | dysentery, bloody dysentery | Huaili, Guanxi, Baha, Lile | 0.2685 | HRC614 |
|  |  |  |  |  |  | crush and apply topically | poison extraction |  |  |  |
| *Angelica decursiva* (Miquel) Franchet & Savatier | ma lian an | Apiaceae | herb | wild | root | crush and apply topically | abscess | Yaoli, Yaozhai, Guanxi, Yaoshan | 0.3241 | HRC93 |
|  |  |  |  |  |  | chew and ingest / decoct in water and take orally | stomach pain, internal injury from a fall |  |  |  |
| *Angiopteris fokiensis* Hieron. |  | Marattiaceae | herb | wild | stem | decoct in water and take orally / cook as porridge | immune boosting, body weakness and soreness | Guanxi, Yaoshan | 0.1111 | HRC926 |
| *Anisomeles indica* (Linnaeus) Kuntze | e fang qu | Lamiaceae | herb | wild | whole plant | decoct in water and take orally | stomach pain, high blood pressure | Guanxi, Yaoshan | 0.1481 | HRC1426 |
|  |  |  |  |  |  | soak in wine and take internally | rheumatism, eczema |  |  |  |
| *Aralia chinensis* Linnaeus | nong mie ba da | Araliaceae | shrub | wild | root | decoct in water and take orally | liver cirrhosis with ascites, stones, carbuncle, vaginal discharge, lumbar sprain, nocturnal emission, rheumatism | Yaoli, Yaozhai, Guanxi | 0.1759 | HRC52 |
| *Ardisia crispa* (Thunberg) A. de Candolle | tong bu gong | Primulaceae | shrub | wild | root, leaf | decoct in water and take orally | sore throat, traumatic injury, excessive cough with sputum, dental pain due to heat, stomach heat pain | Yaoshan | 0.0833 | HRC1419 |
| *Ardisia faberi* Hemsley in F. B. Forbes & Hemsley |  | Primulaceae | herb | wild | whole plant | decoct in water and take orally | cough, pneumonia, stomach pain, cough | Yaoli, Guanxi, Lile | 0.2593 | HRC2 |
| *Ardisia filiformis* E. Walker |  | Primulaceae | shrub | wild | root | soak in wine and take internally | immune boosting | Yaoli, Lile, Yaozhai | 0.1204 | HRC149 |
|  |  |  |  |  |  | crush and apply topically | bone setting |  |  |  |
| *Ardisia gigantifolia* Stapf | zhuo ma zai zhe | Primulaceae | shrub | wild | whole plant | decoct in water and take orally / soak in wine and take internally | immune boosting, hemiplegia | Guanxi | 0.0556 | HRC362 |
| *Ardisia japonica* (Thunberg) Blume | long zha rao | Primulaceae | shrub | wild | whole plant | decoct in water and take orally | tuberculosis | Yaoli, Guanxi, Lile | 0.2685 | HRC172 |
| *Arisaema heterophyllum* Blume | wei bai rao | Araceae | herb | wild | tuber | crush and apply topically | snake bite, hemiplegia, neurodermatitis, mumps in children | Guanxi | 0.0556 | HRC1458 |
| *Artemisia argyi* H. Leveille & Vaniot |  | Asteraceae | herb | wild | leaf | crush and apply topically | hemostasis | Lile, Yaozhai, Yaoli, Guanxi, Yaoshan, Huaili, Lihu | 0.4444 | HRC171 |
| *Artemisia lavandulifolia* Candolle | ya huo | Asteraceae | herb | wild | branches and leaves | decoct in water and wash externally | body itch | Yaoshan, Guanxi | 0.1667 | HRC708 |
| *Asarum caudigerum* Hance |  | Aristolochiaceae | herb | wild | whole plant | cook as porridge | immune boosting | Lile | 0.1019 | HRC526 |
| *Asparagus cochinchinensis* (Loureiro) Merrill | qi jie mei | Asparagaceae | herb | wild | tuber | cook as porridge / soak in wine and take internally | immune boosting | Lile, Guanxi | 0.2593 | HRC144 |
| *Asparagus lycopodineus* (Baker) F. T. Wang & T. Tang | qi jie mei | Asparagaceae | herb | wild | tuber | cook as porridge | immune boosting | Yaoli, Lile, Guanxi | 0.2037 | HRC806 |
| *Aspidopterys glabriuscula* A. Jussieu |  | Malpighiaceae | liana | wild | branches and leaves | crush, extract juice, and apply to affected area | deafness, tinnitus | Guanxi | 0.0463 | HRC236 |
| *Aster indicus* Linnaeus | ya la sai | Asteraceae | herb | wild | whole plant | decoct in water and take orally | high blood pressure, hemostasis | Yaoli, Guanxi | 0.0741 | HRC38 |
|  |  |  |  |  |  | sun-dry, grind to powder, and apply externally / crush and apply topically | bone setting, traumatic injury, carbuncle, wound hemostasis |  |  |  |
| *Aster ageratoides* Turcz. |  | Asteraceae | herb | wild | branches and leaves | decoct in water and take orally | internal injury from a fall | Dongjia | 0.0370 | HRC912 |
| *Asystasia nemorum* Nees in Wallich |  | Acanthaceae | herb | wild | whole plant | decoct in water and take orally | antidote for poison (reduces toxicity of other drugs) | Yaozhai, Guanxi, Yaoli, Lihu | 0.3889 | HRC316 |
| *Atropa belladonna* L. |  | Solanaceae | herb | wild | root | decoct in water and take orally | skin carbuncles | Lile, Huaili, Lihu | 0.2130 | HRC209 |
| *Phanera championii* Benth. | ye guan men | Fabaceae | liana | wild | stem | decoct in water and take orally / soak in wine and take internally | kidney deficiency | Yaoli, Guanxi, Yaozhai, Yaoshan | 0.1944 | HRC272 |
| *Bauhinia* sp. |  | Fabaceae | liana | wild | branches and leaves | decoct in water and wash externally / decoct in water and take orally | traumatic injury, joint pain | Yaoli, Huaili, Guanxi | 0.1204 | HRC539 |
| *Begonia cavaleriei* Lévl. |  | Begoniaceae | herb | wild | stem | decoct in water and take orally | cough | Lile, Guanxi | 0.0648 | HRC147 |
| *Begonia grandis* Dryander | tong bian min lin | Begoniaceae | herb | wild | tuber | soak in wine and take internally | tuberculosis | Yaoli, Huaili, Guanxi, Yaoshan | 0.1759 | HRC91 |
|  |  |  |  |  |  | decoct in water and take orally / decoct in water and take orally | sore throat, coughing blood due to tuberculosis |  |  |  |
| *Belamcanda chinensis* (Linnaeus) Redoute | shou shan hu | Iridaceae | herb | wild or cultivated | tuber | decoct in water and take orally / decoct in water and take orally | tracheitis, bronchitis, stomach bloating | Yaoli, Guanxi, Lile | 0.1204 | HRC191 |
| *Berchemia floribunda* (Wallich) Brongniart | ma guai dan | Rhamnaceae | shrub | wild | old root | soak in wine and take internally | numbness in limbs, traumatic injury | Yaoli | 0.0556 | HRC62 |
| *Bidens pilosa* Linnaeus | e gong | Asteraceae | herb | wild | whole plant | decoct in water and take orally | traumatic injury, liver ascites, dysentery, migraine | Lile, Yaoshan, Yaozhai | 0.2778 | HRC1445 |
|  |  |  |  |  |  | take orally directly | stomach pain |  |  |  |
| *Bidens tripartita* Linnaeus | ai zhong | Asteraceae | herb | wild | branches and leaves | crush and apply topically | traumatic injury, mastitis, snake bite | Guanxi | 0.0648 | HRC690 |
| *Bletilla formosana* (Hayata) Schlechter |  | Orchidaceae | herb | wild | corm | decoct in water and take orally | gastritis, hepatitis | Huaili | 0.1111 | HRC609 |
| *Bletilla striata* (Thunberg) H. G. Reichenbach | zhe he | Orchidaceae | herb | wild | corm | decoct in water and take orally | beauty and whitening, vomiting blood, blood in stool | Yaoli | 0.0833 | HRC44 |
|  |  |  |  |  |  | sun-dry, grind to powder, and take orally | tuberculosis with cavitation |  |  |  |
|  |  |  |  |  |  | crush and apply topically | burn, scald, external injury bleeding |  |  |  |
| *Blumea axillaris* (Lamarck) Candolle |  | Asteraceae | herb | wild | whole plant | decoct in water and take orally | liver cirrhosis | Guanxi | 0.0741 | HRC390 |
| *Blumea riparia* Candolle |  | Asteraceae | herb | wild | whole plant | decoct in water and wash externally | eye pain | Guanxi, Yaoli, Lile | 0.1111 | HRC531 |
| *Boehmeria dolichostachya* W. T. Wang |  | Urticaceae | shrub | wild | leaf | crush and apply topically | snake bite | Dongjia, Yaozhai | 0.0463 | HRC901 |
| *Boehmeria nivea* (Linnaeus) Gaudichaud-Beaupre | ya ga | Urticaceae | tree | wild | leaf | crush and apply topically | syphilis | Guanxi, Yaozhai, Dongjia | 0.2407 | HRC686 |
|  |  |  |  |  |  | decoct in water and wash externally | internal injury, activating blood circulation to remove stasis |  |  |  |
|  |  |  |  |  |  | decoct in water and wash externally | leukorrhea, leukemia |  |  |  |
| *Broussonetia papyrifera* (Linnaeus) L’Heritier ex Ventenat |  | Moraceae | tree | wild or cultivated | latex | apply directly externally | carbuncles, eye swelling after being hit, anti-itch | Lile | 0.0278 | HRC129 |
| *Buddleja officinalis* Maximowicz | bo se | Scrophulariaceae | shrub | wild | root | decoct in water and take orally | pneumonia, jaundice, hepatitis | Yaoli | 0.1019 | HRC79 |
| *Bulbophyllum odoratissimum* (Smith) Lindley | long chao ke | Orchidaceae | herb | wild | whole plant | crush, mix with alcohol, and apply externally | traumatic injury | Yaoshan, Guanxi, Lihu | 0.3241 | HRC1471 |
|  |  |  |  |  |  | decoct in water and take orally | cough with little or no phlegm, coughing blood due to tuberculosis, heavy menstruation |  |  |  |
|  |  |  |  |  |  | stew with meat | chronic osteomyelitis |  |  |  |
| *Caesalpinia sinensis* (Hemsl.) J. E. Vidal |  | Fabaceae | liana | wild | whole plant | decoct in water and wash externally | hemiplegia | Lile | 0.0278 | HRC229 |
| *Callicarpa giraldii* Hesse ex Rehder in Bailey | zu e nuo | Lamiaceae | shrub | wild | branches and leaves | crush and apply topically | shingles | Guanxi | 0.0556 | HRC674 |
| *Callicarpa kwangtungensis* Chun |  | Lamiaceae | shrub | wild | leaf | crush and apply topically | wound anti-inflammation, shingles, carbuncles, abscess | Yaoli | 0.0370 | HRC5 |
| *Callicarpa macrophylla* Vahl | tong pi ci kao | Lamiaceae | shrub | wild | leaf | sun-dry, powder, and apply on affected area | external injury bleeding | Yaoshan, Guanxi | 0.1759 | HRC1425 |
|  |  |  |  |  | root, whole plant | soak in wine and take internally | traumatic injury, rheumatism |  |  |  |
| *Campanumoea javanica* Blume | ya wa | Campanulaceae | herb | wild | tuber | stew with meat | immune boosting | Guanxi | 0.3519 | HRC435 |
| *Canna indica* Linnaeus | lan qiu duan | Cannaceae | herb | cultivated | whole plant | stew with meat | immune boosting, hemostasis, jaundice, excessive leukorrhea, physical weakness | Yaozhai | 0.1204 | HRC788 |
| *Cannabis sativa* Linnaeus |  | Cannabinaceae | herb | cultivated | whole plant | decoct in water and wash externally | unblocking channels, paralysis | Dongjia | 0.0370 | HRC888 |
| *Canscora lucidissima* (H. Leveille & Vaniot) Handel-Mazzetti | chen xin mi | Gentianaceae | herb | wild | whole plant | decoct in water and take orally | antral gastritis, jaundice, hepatitis, stomach pain | Yaoli, Yaoshan | 0.2500 | HRC28 |
|  |  |  |  |  |  | crush and apply topically | traumatic injury |  |  |  |
| *Capsella bursa-pastoris* (Linnaeus) Medikus | e ai rao | Brassicaceae | herb | wild | whole plant | decoct in water and take orally | high blood pressure, diarrhea, clear heat, vomiting blood, heavy menstruation | Yaoshan | 0.0278 | HRC1207 |
| *Cardiocrinum giganteum* (Wallich) Makino |  | Liliaceae | herb | wild | root | chew and ingest | throat obstruction | Guanxi, Yaozhai | 0.0926 | HRC366 |
|  |  |  |  |  |  | decoct in water and take orally | stomach bloating, loss of appetite |  |  |  |
| *Carex baccans* Nees in Wight |  | Cyperaceae | herb | wild | whole plant | decoct in water and take orally | stones, connecting tendons, cramps | Yaoli | 0.0278 | HRC180 |
| *Carex* sp. |  | Cyperaceae | herb | wild | whole plant | decoct in water and wash externally | body aches | Guanxi, Yaozhai | 0.0833 | HRC650 |
| *Carpesium abrotanoides* Linnaeus |  | Asteraceae | herb | wild | leaf | crush and apply topically | scabies, traumatic injury, unidentified swelling and infection | Yaoli, Guanxi, Yaozhai | 0.2593 | HRC21 |
|  |  |  |  |  |  | take orally directly | diarrhea |  |  |  |
| *Carpesium cernuum* Linnaeus |  | Asteraceae | herb | wild | whole plant | take orally directly / decoct in water and take orally | clear heat and detoxify, stomach pain | Yaozhai, Lile, Guanxi | 0.1389 | HRC115 |
| *Cassytha filiformis* Linnaeus |  | Lauraceae | liana | wild | whole plant | decoct in water and take orally | malaria, high fever, jaundice, hepatitis | Yaoli, Lile, Guanxi | 0.1667 | HRC245 |
| *Castanea mollissima* Blume | bi yi | Fagaceae | tree | cultivated | pericarp of chestnut fruit | decoct in water and wash externally | body itch | Guanxi | 0.0463 | HRC684 |
| *Cayratia japonica* (Thunberg) Gagnepain | mo mi ji | Vitaceae | liana | wild | root | decoct in water and take orally | epilepsy | Yaoli, Guanxi | 0.1667 | HRC130 |
| *Celosia cristata* Linnaeus | ya niu | Amaranthaceae | herb | cultivated | whole plant | soak in wine and take internally / decoct in water and take orally | heavy menstruation, menorrhagia | Lile, Guanxi | 0.0556 | HRC300 |
| *Centella asiatica* (Linnaeus) Urban | e de liu zhui | Apiaceae | herb | wild | whole plant | decoct in water and take orally | cloudy urine, sore throat, dysentery, red and swollen eyes | Guanxi, Yaozhai, Yaoshan | 0.2130 | HRC1232 |
|  |  |  |  |  |  | crush, mix with alcohol, and apply externally | traumatic injury, ulcer swelling and pain |  |  |  |
| *Epaltes australis* Less. | e ji mu nao | Asteraceae | herb | wild | whole plant | crush and apply topically | rhinitis, psoriasis | Yaoshan, Guanxi, Yaozhai | 0.1759 | HRC1453 |
|  |  |  |  |  |  | decoct in water and take orally | whooping cough, oral ulcers, child diarrhea |  |  |  |
| *Ceropegia longifolia* Wallich |  | Apocynaceae | liana | wild | tuber | cook with pork bones | kidney nourishment, qi boosting | Yaozhai | 0.0278 | HRC189 |
| *Chenopodium album* Linnaeus |  | Amaranthaceae | herb | wild | branches and leaves | crush, extract juice, and take orally | hookworm poisoning treatment | Yaoli | 0.0185 | HRC70 |
| *Chimonanthus praecox* (Linnaeus) Link | gei ji wei ai | Calycanthaceae | shrub | wild | flower bud | soak in water and take orally | sore throat, abdominal pain | Yaoshan | 0.0556 | HRC1205 |
|  |  |  |  |  | root | soak in wine and take orally | traumatic injury |  |  |  |
|  |  |  |  |  | leaf | soak in vege-oil for a week, then apply externally | scald |  |  |  |
| *Chloranthus holostegius* (Handel-Mazzetti) Pei & Shan | zhu lian long | Chloranthaceae | herb | wild | whole plant | soak in wine and take internally | back pain | Guanxi | 0.0278 | HRC448 |
| *Chromolaena odorata* (Linnaeus) R. M. King & H. Robinson |  | Asteraceae | herb | wild | whole plant | decoct in water and take orally | joint loosening, hemiplegia, phlegm cough, chronic dysentery | Yaoli, Yaozhai, Guanxi, Dongjia, Lile | 0.2685 | HRC219 |
|  |  |  |  |  |  | crush and apply topically | swelling reduction, unidentified swelling and infection |  |  |  |
| *Chrysanthemum indicum* Linnaeus | ya ho | Asteraceae | herb | wild | whole plant | crush and apply topically | rheumatoid arthritis | Yaoli, Huaili, Lile, Guanxi | 0.3519 | HRC80 |
|  |  |  |  |  |  | decoct in water and take orally | clear heat and relieve fire |  |  |  |
| *Cinnamomum camphora* (Linnaeus) J. Presl in Berchtold & J. Presl | tong wei gao | Lauraceae | tree | wild or cultivated | stem | soak in wine and take internally | alcohol detoxification, dispel rheumatism | Yaoshan, Yaoli | 0.1204 | HRC30 |
|  |  |  |  |  |  | decoct in water and take orally | anti-inflammatory, hernia, skin eruption |  |  |  |
| *Cirsium arvense* var. *integrifolium* Wimmer & Grabowski | e wen jian fan bo | Asteraceae | herb | wild | whole plant | crush and apply topically | hemostasis | Yaoshan | 0.0741 | HRC1448 |
|  |  |  |  |  |  | decoct in water and take orally | excessive menstruation in women, cough |  |  |  |
| *Cirsium japonicum* (Thunb.) Fisch. ex DC. |  | Asteraceae | herb | wild | root | cook with pork intestines | hemorrhoids, diarrhea | Huaili, Yaozhai | 0.0648 | HRC598 |
| *Clausena dunniana* H. Leveille |  | Rutaceae | shrub | wild | branches and leaves | decoct in water and take orally | joint loosening, pain | Lile | 0.0463 | HRC224 |
| *Clematis chinensis* Osbeck |  | Ranunculaceae | liana | wild | root | decoct in water and wash externally / decoct in water and take orally | gout | Yaozhai, Guanxi | 0.1111 | HRC184 |
| *Clematis uncinata* Champion ex Bentham |  | Ranunculaceae | liana | wild | whole plant | decoct in water and wash externally | numbness in hands and feet | Baha | 0.0463 | HRC542 |
| *Clerodendrum bungei* Steudel | long qi mo | Lamiaceae | shrub | wild | inflorescence | crush and apply topically | hemorrhoids, skin diseases, anti-itch | Huaili, Yaoshan, Baha, Lile | 0.4167 | HRC200 |
|  |  |  |  |  |  | decoct in water and take orally | high blood pressure, antral gastritis, rectal cancer |  |  |  |
|  |  |  |  |  |  | decoct in water and wash externally | skin itch, snake bite |  |  |  |
| *Clerodendrum chinense* var. *simplex* (Moldenke) S. L. Chen |  | Lamiaceae | shrub | wild | root | decoct in water and wash externally | urticaria | Yaoli | 0.0556 | HRC88 |
| *Cocculus laurifolius* Candolle |  | Menispermaceae | shrub | wild | branches and leaves | decoct in water and wash externally | swelling and pain in hands and feet, fatigue | Dongjia | 0.0463 | HRC264 |
| *Codonopsis tubulosa* Komarov | e wu | Campanulaceae | herb | wild | root | stew with meat | physical weakness, weak cough, postpartum low milk supply, childhood enuresis | Yaoshan | 0.1111 | HRC1443 |
|  |  |  |  |  |  | decoct in water and take orally | childhood enuresis |  |  |  |
| *Coix lacryma-jobi* Linnaeus | ai jie | Poaceae | herb | cultivated | seed | decoct in water and take orally / decoct in water and take orally | stones, coughing thick phlegm, blurred vision, edema | Guanxi | 0.0556 | HRC283 |
|  |  |  |  |  |  | steam with pork liver | excessive vaginal discharge in women |  |  |  |
| *Commelina diffusa* N. L. Burm. | e tou fo | Commelinaceae | herb | wild | whole plant | decoct in water and take orally | child fever, urinary discomfort, edema, high blood pressure, dizziness, dysentery | Lile, Yaozhai, Yaoshan | 0.1481 | HRC1459 |
|  |  |  |  |  |  | crush and apply topically | unidentified swelling and infection |  |  |  |
| *Cordyline fruticosa* (Linnaeus) A. Chevalier |  | Asparagaceae | herb | cultivated | whole plant | soak in wine and take internally | traumatic injury | Yaozhai | 0.0556 | HRC467 |
| *Coriandrum sativum* Linnaeus | e ma yu qi | Apiaceae | herb | cultivated | whole plant | decoct in water and take orally | cold and flu, poor appetite, indigestion, chest tightness, prolapse, fungal poisoning | Yaoshan | 0.0278 | HRC1236 |
| *Coriaria nepalensis* Wallich |  | Coriariaceae | shrub | wild | branches and leaves | crush and apply topically | anti-inflammatory, insecticidal and anti-itch, pain relief, external traumatic injury | Yaoli, Guanxi, Dongjia | 0.1204 | HRC627 |
| *Corydalis saxicola* Bunting |  | Papaveraceae | herb | wild | whole plant | decoct in water and take orally | tuberculosis, kidney stones, appendicitis, rheumatic bone pain | Yaozhai, Lile, Guanxi | 0.2593 | HRC109、HRC271 |
|  |  |  |  |  |  | soak in wine and take internally | stomach pain, rheumatic bone pain, menorrhagia |  |  |  |
| *Costus speciosus* (J. Konig) Smith | gu shou | Costaceae | herb | wild | tuber | stew with pig feet | immune boosting | Guanxi, Baha, Yaozhai | 0.1481 | HRC692 |
| *Crassocephalum crepidioides* (Bentham) S. Moore |  | Asteraceae | herb | wild | fruit | wrap the affected area with the fruit | hemostasis | Yaoli, Yaozhai | 0.1204 | HRC126 |
| *Crataegus cuneata* Siebold & Zuccarini | tong guo la | Rosaceae | shrub | wild | fruit | decoct in water and take orally | food stagnation and bloating, diarrhea, amenorrhea, postpartum abdominal pain in women | Yaoshan | 0.1481 | HRC1214 |
| *Crepidiastrum denticulatum* (Houttuyn) Pak & Kawano |  | Asteraceae | herb | wild | tender branches and leaves | cooked thoroughly and take orally | lower blood pressure | Dongjia, Yaozhai | 0.0556 | HRC909 |
| *Crinum asiaticum* var. *sinicum* (Roxburgh ex Herbert) Baker |  | Amaryllidaceae | herb | cultivated | whole plant | heat and apply externally | traumatic injury | Yaozhai, Guanxi, Yaoshan | 0.1667 | HRC333 |
|  |  |  |  |  |  | crush and apply topically | snake venom antidote |  |  |  |
|  |  |  |  |  |  | decoct in water and take orally | indigestion |  |  |  |
|  |  |  |  |  |  | soak in wine and take internally | witchcraft poison antidote, bloody dysentery |  |  |  |
| *Cryptotaenia japonica* Hasskarl |  | Apiaceae | herb | wild | whole plant | soak in wine and take internally | joint pain, headache | Lile | 0.0463 | HRC505 |
| *Cucumis sativus* Linnaeus |  | Cucurbitaceae | liana | cultivated | tender leaf | take orally directly | diarrhea | Yaozhai, Lile | 0.1852 | HRC336 |
| *Cunninghamia lanceolata* (Lambert) Hooker | luo ji | Cupressaceae | tree | cultivated | leaf | crush and apply topically | snake bite | Guanxi | 0.0278 | HRC696 |
| *Curculigo orchioides* Gaertner |  | Hypoxidaceae | herb | wild | tuber | soak in wine and take internally | aphrodisiac | Yaoli, Yaozhai, Guanxi, Yaoshan | 0.4444 | HRC54 |
| *Curcuma aromatica* Salisbury |  | Zingiberaceae | herb | cultivated | whole plant | decoct in water and wash externally | unblocking channels, paralysis | Guanxi | 0.0556 | HRC889 |
| *Curcuma longa* Linnaeus | gang | Zingiberaceae | herb | wild or cultivated | tuber | crush and apply topically | traumatic injury, remove necrotic tissue and promote granulation | Yaoli, Guanxi, Baha, Yaozhai | 0.3519 | HRC552 |
|  |  |  |  |  |  | decoct in water and wash externally / decoct in water and take orally | jaundice hepatitis, hepatitis B |  |  |  |
| *Curcuma phaeocaulis* Valeton |  | Zingiberaceae | herb | cultivated | tuber | decoct in water and take orally | jaundice, hepatitis, appendicitis | Guanxi, Lile, Baha, Yaozhai | 0.1389 | HRC284 |
|  |  |  |  |  |  | crush and apply topically | back pain, bone setting, traumatic injury |  |  |  |
| *Cuscuta chinensis* Lamarck |  | Convolvulaceae | liana | cultivated | whole plant | crush and apply topically | tendon connecting | Guanxi | 0.0278 | HRC929 |
| *Cyanthillium cinereum* (L.) H. Rob. | e fu niu | Asteraceae | herb | wild | whole plant | decoct in water and take orally | external heat causing fever, jaundice, traumatic injury | Guanxi, Huaili, Yaoshan | 0.1481 | HRC1451 |
| *Cyclea hypoglauca* (Schauer) Diels in Engler | bai jie teng | Menispermaceae | liana | wild | whole plant | decoct in water and take orally | poisoning, stomach pain, antral gastritis, stomach pain, stones | Yaoli, Lile, Yaozhai, Guanxi | 0.2963 | HRC176 |
|  |  |  |  |  |  | decoct in water and wash externally | hemiplegia |  |  |  |
| *Cyclea racemosa* Oliv. | mu gu biao rao | Menispermaceae | liana | wild | root | soak in water and take orally | stomach pain, hiccup | Yaoshan | 0.0463 | HRC1202 |
|  |  |  |  |  |  | crush and apply topically | snake bite |  |  |  |
| *Cyclosorus dentatus* (Forssk.) Ching | zong | Thelypteridaceae | herb | wild | whole plant | decoct in salty water and wash externally | activate blood circulation, remove blood stasis | Dongjia | 0.0278 | HRC895 |
| *Cymbopogon citratus* (Candolle) Stapf | xia yi | Poaceae | herb | cultivated | whole plant | decoct in water and take orally | cough, sore throat | Guanxi, Yaozhai | 0.2315 | HRC711 |
| *Vincetoxicum pycnostelma* Kitag. |  | Apocynaceae | herb | wild | whole plant | crush and apply topically | snake bite poisoning, bee sting, unidentified swelling and infection | Guanxi | 0.0278 | HRC927 |
| *Cynanchum wilfordii* (Maximowicz) J. D. Hooker | zhu nu se | Apocynaceae | liana | wild | tuber | decoct in water and take orally | aid digestion, stomach pain, aid digestion | Guanxi, Yaozhai | 0.1481 | HRC418 |
|  |  |  |  |  |  | steam with meat | lactation stimulation |  |  |  |
| *Cyperus rotundus* Linnaeus | e jie yi | Cyperaceae | herb | wild | whole plant | sun-dry and decoct in water and take orally | stomach pain | Yaoli | 0.0556 | HRC620 |
| *Dahlia pinnata* Cav. |  | Asteraceae | herb | cultivated | tuber | stew with meat | immune boosting | Guanxi | 0.0556 | HRC694 |
| *Datura stramonium* Linnaeus | e long yan | Solanaceae | herb | cultivated | leaf | decoct in water and wash externally | rheumatic joint pain, traumatic injury | Yaoshan | 0.0463 | HRC1429 |
|  |  |  |  |  | root bark | crush and wash externally | psoriasis |  |  |  |
| *Debregeasia longifolia* (N. L. Burman) Weddell in Candolle |  | Urticaceae | shrub | wild | leaf | crush and apply topically | bone setting | Dongjia | 0.0370 | HRC902 |
| *Decaspermum gracilentum* (Hance) Merrill & L. M. Perry |  | Myrtaceae | tree | wild | leaf | chew and apply topically | gum swelling and pain | Dongjia | 0.0741 | HRC261 |
| *Dichondra micrantha* Urban | e de le zhui | Convolvulaceae | herb | wild | whole plant | decoct in water and take orally | jaundice hepatitis, urinary difficulties, menstrual irregularities, fever | Lile, Guanxi, Yaoshan | 0.1111 | HRC1424 |
|  |  |  |  |  |  | crush and apply topically | traumatic injury, unidentified swelling and infection |  |  |  |
| *Dicliptera chinensis* (Linnaeus) Jussieu | e qin zhui | Acanthaceae | herb | wild | whole plant | decoct in water and take orally | clear heat and relieve fire, furuncle | Yaozhai, Yaoli, Guanxi | 0.2315 | HRC1433 |
|  |  |  |  |  |  | crush and apply topically | unidentified swelling and infection |  |  |  |
| *Dicranopteris pedata* (Houtt.) Nakaike | pa jia | Gleicheniaceae | herb | wild | spore | decoct in water and wash externally | beauty and whitening | Yaoli | 0.0370 | HRC43 |
| *Dioscorea bulbifera* Linnaeus | wei yo | Dioscoreaceae | liana | wild | haustorium | decoct in water and take orally | unidentified swelling and infection, snake bite, hyperthyroidism | Guanxi, Yaoshan, Yaozhai | 0.1481 | HRC706 |
| *Dioscorea cirrhosa* Loureiro | nei re | Dioscoreaceae | liana | wild | tuber | crush and apply topically | hemostasis | Guanxi, Yaozhai, Yaoli | 0.1481 | HRC726 |
|  |  |  |  |  |  | decoct in water and take orally | bloody dysentery |  |  |  |
| *Dioscorea persimilis* Prain et Burkill | du sha | Dioscoreaceae | liana | cultivated | root | decoct in water and take orally | spleen deficiency with poor appetite, pale complexion, thin physique, childhood diarrhea, nocturnal emission | Yaozhai, Yaoshan, Huaili | 0.3889 | HRC1468 |
| *Dioscorea subcalva* Prain & Burkill | du lv | Dioscoreaceae | liana | wild or cultivated | tuber | decoct in water and take orally | tuberculosis | Yaozhai, Huaili | 0.0556 | HRC1469 |
|  |  |  |  |  |  | crush and apply topically | chapped skin, carbuncle |  |  |  |
| *Dipsacus asper* Wallich ex C. B. Clarke | jiu ge tao | Dipsacaceae | herb | wild | root | soak in wine and take internally | rheumatic pain, traumatic injury, back pain, unidentified swelling and infection | Yaozhai, Guanxi, Yaoshan | 0.1759 | HRC1440 |
|  |  |  |  |  |  | crush and apply topically | unidentified swelling and infection |  |  |  |
| *Disporopsis pernyi* (Hua) Diels | huang jiao ji | Liliaceae | herb | wild | whole plant | crush and apply topically | snake venom antidote | Yaozhai, Yaoli, Lihu, Lile | 0.4722 | HRC400 |
|  |  |  |  |  |  | soak in wine and take internally / decoct in water and take orally | witchcraft poison antidote |  |  |  |
| *Disporum cantoniense* (Loureiro) Merrill | zhong yao | Colchicaceae | herb | wild | leaf | stew with chicken / decoct in water and take orally / crush, mix with alcohol, and apply externally | antidote for poisoning, physical weakness, numbness in hands and feet, weak cough, fracture | Lile | 0.0463 | HRC248 |
| *Drynaria roosii* Nakaike | duo ruo wei ai | Polypodiaceae | herb | wild | tuber | soak in wine and take internally | back pain, joint pain | Lile, Guanxi, Huaili, Yaoshan | 0.2963 | HRC429 |
|  |  |  |  |  |  | crush, extract juice, boil, and use for brushing teeth | gum swelling and pain |  |  |  |
| *Duchesnea indica* (Andrews) Focke in Engler & Prantl | gei gao | Rosaceae | herb | wild | fruit | crush, mix with alcohol, and apply externally | traumatic injury, unidentified swelling and infection | Yaoshan | 0.0463 | HRC1211 |
|  |  |  |  |  |  | decoct in water and take orally | eczema, dysentery, high blood pressure with dizziness |  |  |  |
| *Duhaldea cappa* (Buchanan-Hamilton ex D. Don) Pruski & Anderberg | bai mian feng | Asteraceae | herb | wild | whole plant | decoct in water and take orally | unexplained edema, liver cirrhosis | Guanxi | 0.0278 | HRC187 |
| *Dysosma versipellis* (Hance) M. Cheng ex T. S. Ying |  | Berberidaceae | herb | wild | tuber | decoct in water and take orally | contraception | Guanxi | 0.0278 | HRC458 |
| *Eclipta prostrata* (Linnaeus) Linnaeus | e bei duo wei nao | Asteraceae | herb | wild | whole plant | decoct in water and take orally | premature greying, excessive menstruation in women, diarrhea and dysentery | Yaoshan, Huaili, Yaoli, Yaozhai | 0.2222 | HRC1449 |
|  |  |  |  |  |  | crush and apply topically | hemostasis, rotten feet |  |  |  |
| *Edgeworthia chrysantha* Lindley |  | Thymelaeaceae | shrub | cultivated | whole plant | crush and apply topically | bone setting | Huaili | 0.0556 | HRC635 |
| *Elephantopus scaber* Linnaeus |  | Asteraceae | herb | wild | whole plant | decoct in water and take orally | clear heat and relieve fire | Yaozhai | 0.1204 | HRC873 |
| *Eleusine indica* (Linnaeus) Gaertner |  | Poaceae | herb | wild | whole plant | decoct in water and take orally | jaundice, hepatitis | Guanxi | 0.0741 | HRC213 |
| *Eleutherine plicata* Herb. | jue nie yi | Iridaceae | herb | cultivated | corm | soak in wine and take internally | blood tonic, immune boosting | Guanxi | 0.0278 | HRC702 |
| *Eleutherococcus nodiflorus* (Dunn) S. Y. Hu |  | Araliaceae | shrub | cultivated | root | soak in wine and take internally | traumatic injury, weakness in hands and feet | Yaoli, Yaozhai, Huaili, Guanxi | 0.3519 | HRC87 |
|  |  |  |  |  |  | crush and apply topically | swelling reduction and pain relief, bone setting |  |  |  |
| *Eleutherococcus trifoliatus* (Linnaeus) S. Y. Hu |  | Araliaceae | liana | cultivated | root | stew with meat / soak in wine and take internally | weakness and softness, osteonecrosis | Yaoli, Huaili | 0.0833 | HRC642 |
| *Elsholtzia argyi* H. Leveille | yong jie | Lamiaceae | herb | wild | branches and leaves | decoct in water and wash externally | vitiligo | Guanxi | 0.0556 | HRC441 |
| *Emilia sonchifolia* (L.) DC. |  | Asteraceae | herb | wild | whole plant | decoct in water and take orally | internal heat, anti-inflammatory | Guanxi, Yaoli, Yaoshan | 0.2593 | HRC304 |
| *Engelhardia spicata* var. *integra* (Kurz) Grierson & Long |  | Juglandaceae | shrub | wild | stem, root | crush and apply topically | traumatic injury | Guanxi | 0.0278 | HRC305 |
|  |  |  |  |  |  | decoct in water and wash externally | paralysis |  |  |  |
| *Epimedium sagittatum* (Siebold & Zuccarini) Maximo-wicz | jiao yang huo | Berberidaceae | herb | wild | fruit, root | soak in wine and take internally | aphrodisiac, kidney nourishment | Yaozhai, Yaoshan, Guanxi, Lile | 0.3056 | HRC170 |
| *Equisetum diffusum* D. Don |  | Equisetaceae | herb | wild | whole plant | decoct in water and take orally | diuretic, urinary obstruction | Guanxi | 0.0648 | HRC378 |
| *Eriobotrya japonica* (Thunberg) Lindley | bi bo le | Rosaceae | tree | cultivated | leaf, flower | decoct in water and take orally | cough, nausea and vomiting | Yaoshan, Huaili, Lihu, Guanxi, Lile, Yaoli, Yaozhai | 0.5370 | HRC204 |
|  |  |  |  |  | leaf | crush and apply topically | bone setting |  |  |  |
|  |  |  |  |  | root | soak in wine and take internally | rheumatism |  |  |  |
| *Eucommia ulmoides* Oliver |  | Eucommiaceae | tree | cultivated | bark | crush and apply topically | traumatic injury, unidentified swelling and pain | Yaoli, Guanxi, Yaozhai, Lihu, Yaoshan | 0.4537 | HRC353 |
|  |  |  |  |  |  | decoct in water and take orally | bone setting, tendon connecting |  |  |  |
| *Eupatorium fortunei* Turczaninow |  | Asteraceae | herb | wild | whole plant | crush and take orally | external injury | Yaozhai, Guanxi, Huaili, Baha | 0.1111 | HRC255 |
|  |  |  |  |  |  | crush and apply topically | throat obstruction |  |  |  |
|  |  |  |  |  |  | soak in wine and take internally / decoct in water and take orally | witchcraft poison antidote, knife injury, uterine traumatic injury |  |  |  |
| *Euphorbia esula* Linnaeus |  | Euphorbiaceae | herb | wild | leaf | crush and apply topically | skin itching | Yaozhai | 0.0370 | HRC168 |
| *Euphorbia lathyris* Linnaeus |  | Euphorbiaceae | herb | cultivated | fruit | take orally | constipation | Guanxi | 0.0463 | HRC457 |
| *Fagopyrum dibotrys* (D. Don) H. Hara | e hong | Polygonaceae | herb | cultivated | rhizome | crush, mix with alcohol, and apply externally | traumatic injury | Yaoshan | 0.0741 | HRC1192 |
|  |  |  |  |  |  | decoct in water and take orally | carbuncle pain, sore throat, menstrual irregularities, stomach pain |  |  |  |
| *Ficus sarmentosa* Buch.-Ham. ex Sm. var. *lacrymans* (Lév.) Corner |  | Moraceae | liana | wild | leaf | decoct and use as a mouthwash | toothache | Huaili | 0.0278 | HRC637 |
| *Ficus tikoua* Bureau | ji wo die | Moraceae | liana | wild | whole plant | decoct in water and take orally | malaria, alcohol detox, toothache, abscess, bone setting, lactation stimulation, alcohol poisoning | Yaoli, Guanxi, Lile, Yaozhai | 0.2037 | HRC419 |
| *Flueggea virosa* (Roxburgh ex Willdenow) Voigt |  | Phyllanthaceae | shrub | wild | stem | decoct in water and take orally | high blood sugar, diabetes | Guanxi | 0.0463 | HRC370 |
| *Galium spurium* Linnaeus | e mei tao | Rubiaceae | herb | wild | whole plant | decoct in water and take orally | bacterial dysentery, painful urination | Dongjia, Yaoshan | 0.1667 | HRC1435 |
|  |  |  |  |  |  | crush and apply topically | hemostasis, swelling and poison reduction |  |  |  |
| *Gaultheria leucocarpa* var. *yunnanensis* (Franchet) T. Z. Hsu & R. C. Fang | luo gong gou | Ericaceae | shrub | wild | branches and leaves | soak in alcohol and drink / decoct in water and wash externally | paralysis, joint pain, unidentified swelling and infection | Guanxi | 0.0741 | HRC376 |
| *Gelsemium elegans* (Gardner & Champion) Bentham |  | Gelsemiaceae | liana | wild | root | decoct in water and take orally | late-stage gastric cancer, intestinal cancer | Yaoli | 0.0185 | HRC69 |
| *Gentiana rhodantha* Franchet in F. B. Forbes & Hems ley | luo la lao | Gentianaceae | herb | wild | whole plant | decoct in water and take orally | clear internal heat, jaundice, lung heat cough, short and red urine | Yaoshan | 0.0463 | HRC1423 |
|  |  |  |  |  |  | crush and apply topically | unidentified swelling and infection, minor burn injury |  |  |  |
| *Geranium nepalense* Sweet | e ka liao cha | Geraniaceae | herb | wild | whole plant | decoct in water and take orally | dysentery, cough, external injury bleeding | Yaoshan, Yaozhai | 0.0833 | HRC1222 |
|  |  |  |  |  |  | soak in wine and take internally | rheumatic pain, male impotence |  |  |  |
| *Gerbera piloselloides* (L.) Cass. | e jiu bo sa | Asteraceae | herb | wild | whole plant | decoct in water and take orally | cough, traumatic injury, pediatric indigestion, painful urination | Yaozhai, Guanxi, Huaili, Yaoshan | 0.2870 | HRC1450 |
| *Geum japonicum* Thunberg var. *chinense* F. Bolle | jio ka | Rosaceae | herb | wild | whole plant | decoct in water and take orally | cerebral thrombosis | Guanxi | 0.0833 | HRC313 |
| *Gladiolus gandavensis* Van Houtte | suan pan guo | Iridacea | herb | wild | whole plant | decoct in water and take orally | sore throat, cough | Guanxi, Yaozhai, Lihu, Yaoli, Yaoshan | 0.1667 | HRC355 |
| *Glechoma longituba* (Nakai) Kuprianova | e pi ha zhui | Lamiaceae | herb | wild | whole plant | decoct in water and take orally | stones, edema, menstrual irregularities | Guanxi, Yaozhai, Lihu, Yaoli, Yaoshan | 0.2593 | HRC332 |
|  |  |  |  |  |  | crush and apply topically | skin itching, traumatic injury |  |  |  |
| *Glochidion puberum* (Linnaeus) Hutchinson in Sargent | tong ke gu gua | Phyllanthaceae | shrub | wild | branches and leaves | decoct in water and take orally | diarrhea, witchcraft poison antidote | Guanxi | 0.0556 | HRC287 |
|  |  |  |  |  |  | crush and apply topically | scald |  |  |  |
|  |  |  |  |  |  | chew and ingest | gum swelling and pain |  |  |  |
|  |  |  |  |  |  | decoct in water and wash externally | traumatic injury |  |  |  |
| *Gonostegia hirta* (Blume) Miquel | zhan zhan yao | Urticaceae | herb | wild | branches and leaves | crush and apply topically | hemostasis | Yaoli | 0.0278 | HRC48 |
| *Goodyera schlechtendaliana* H. G. Reichenbach | e hao ai | Orchidaceae | herb | wild | whole plant | decoct in water and take orally | cough, coughing blood, loss of appetite | Lile, Guanxi, Huaili, Dongjia | 0.2778 | HRC1472 |
| *Gouania leptostachya* Candolle |  | Rhamnaceae | herb | wild | branches and leaves | decoct in water and take orally | frequent nocturnal urination, pediatric indigestion | Guanxi | 0.0463 | HRC385 |
| *Gynostemma pentaphyllum* (Thunberg) Makino |  | Cucurbitaceae | herb | wild | whole plant | decoct in water and take orally | stomach pain, angina, witchcraft poison antidote, liver cirrhosis | Guanxi, Yaozhai, Lile, Yaoli, Yaoshan | 0.4630 | HRC125 |
|  |  |  |  |  |  | crush and apply topically | muscle soreness |  |  |  |
| *Gynura japonica* (Thunberg) Juel | e zhai ta | Asteraceae | herb | wild | whole plant | crush and apply topically | traumatic injury, hemostasis, insect sting injury | Yaozhai, Yaoshan | 0.1389 | HRC328 |
| *Habenaria dentata* (Swartz) Schlechter | suan shen cao | Orchidaceae | herb | wild | corm | stew with pork kidney | kidney nourishment, aphrodisiac | Yaozhai, Huaili, Yaoli | 0.1852 | HRC188 |
|  |  |  |  |  |  | crush and apply topically | snake bite |  |  |  |
| *Hedera nepalensis* K. Koch var. *sinensis* (Tobler) Rehder | san jiao feng | Araliaceae | liana | wild | whole plant | decoct in water and wash externally | rheumatism, unblocking channels, paralysis | Yaoli | 0.0556 | HRC13 |
| *Hedychium coronarium* J. Konig in Retzius | du shou | Zingiberaceae | herb | wild | fruit | stew with meat | physical weakness with night sweats, lack of strength | Baha | 0.0463 | HRC1470 |
|  |  |  |  |  | root | decoct in water and take orally | rheumatic bone pain, stomach bloating and fullness |  |  |  |
| *Hedychium* sp. |  | Zingiberaceae | herb | wild | tuber | stew with meat | stomach pain, gastritis, traumatic injury | Baha | 0.0463 | HRC555 |
| *Hedyotis hedyotidea* (Candolle) Merrill | geng biao sou | Rubiaceae | herb | wild | whole plant | crush, extract juice, and apply to affected area | rhinitis | Guanxi | 0.0278 | HRC673 |
| *Houttuynia cordata* Thunberg | ya zha | Saururaceae | herb | wild | whole plant | decoct in water and take orally | tuberculosis cough, night sweats, unidentified swelling and infection, sore throat, dysentery, urinary difficulties | Yaoli, Guanxi, Yaozhai, Yaoshan, Lile | 0.2778 | HRC111 |
| *Humulus scandens* (Loureiro) Merrill | e ka chao mo | Cannabinaceae | herb | wild | whole plant | powdered and apply externally | sore | Yaozhai | 0.0556 | HRC1189 |
|  |  |  |  |  |  | decoct in water and take orally | diarrhea, urinary stones, tuberculosis fever, skin itching, cough with thick sputum |  |  |  |
| *Hydrocotyle nepalensis* Hooker |  | Araliaceae | herb | wild | whole plant | crush and apply topically | snake bite | Lile, Yaozhai | 0.0926 | HRC259 |
|  |  |  |  |  |  | decoct in water and take orally | cough, insecticide |  |  |  |
| *Hydrocotyle sibthorpioides* Lamarck | e dou liu zhui rao | Araliaceae | herb | wild | whole plant | decoct in water and take orally | jaundice hepatitis, coughing blood, pediatric indigestion, urinary stones, red and swollen eyes | Lile, Yaoshan | 0.1019 | HRC1235 |
|  |  |  |  |  |  | crush and apply topically | athlete's foot (tinea pedis) |  |  |  |
| *Hypericum japonicum* Thunberg in Murray |  | Hypericaceae | herb | wild | whole plant | crush and apply topically | eye pain, cataract | Guanxi, Yaozhai, Yaoli | 0.1852 | HRC118 |
|  |  |  |  |  |  | decoct in water and take orally | urinary tract infection |  |  |  |
| *Hypericum sampsonii* Hance | e long pu nuo | Hypericaceae | herb | wild | whole plant | decoct in water and wash externally | witchcraft poison antidote | Lile, Yaoshan | 0.0556 | HRC514 |
|  |  |  |  |  |  | crush and apply topically | joint dislocation, swelling reduction and pain relief |  |  |  |
| *Illigera rhodantha* Hance | hua mei rou | Hernandiaceae | liana | wild | stem, root | decoct in water and take orally / decoct in water and wash externally | unblocking channels, rheumatism, paralysis, traumatic injury | Yaoli, Yaoli, Guanxi, Lile, Yaozhai, Huaili, Dongjia | 0.3519 | HRC10 |
| *Impatiens balsamina* Linnaeus | zu nao ai mi | Balsaminaceae | herb | wild | seed | take orally directly | postpartum retention of placenta (placental retention) | Yaoli, Guanxi, Yaozhai, Lile, Lihu, Yaoshan | 0.3611 | HRC206 |
|  |  |  |  |  |  | decoct in water and wash externally | rheumatic bone pain |  |  |  |
|  |  |  |  |  |  | decoct in water and take orally | traumatic injury, amenorrhea, snake bite |  |  |  |
| *Imperata cylindrica* (Linnaeus) Raeuschel |  | Poaceae | herb | wild | root | decoct in water and take orally | leg cramp | Yaozhai, Baha | 0.0741 | HRC196 |
|  |  |  |  |  |  | decoct in water and take orally | stomach pain |  |  |  |
| *Indigofera bungeana* Walpers |  | Fabaceae | shrub | wild | leaf | crush and apply topically | skin carbuncles, itching, aid in wound healing | Yaoli | 0.0278 | HRC103 |
| *Indigofera esquirolii* H. Leveille | dong | Fabaceae | shrub | wild | leaf | crush and apply topically | skin carbuncles, itching | Yaoli | 0.0185 | HRC101 |
| *Ipomoea batatas* (Linnaeus) Lamarck | yan du | Convolvulaceae | liana | cultivated | leaf | crush and apply topically | centipede bite | Lile | 0.0463 | HRC705 |
| *Ipomoea nil* (Linnaeus) Roth |  | Convolvulaceae | liana | wild | seed | chew and ingest | liver cirrhosis with ascites | Yaoli | 0.0556 | HRC47 |
| *Iris japonica* Thunberg |  | Iridaceae | herb | wild | rhizome | crush and apply topically | bone setting | Baha, Yaozhai, Guanxi | 0.1759 | HRC549 |
|  |  |  |  |  |  | decoct in water and take orally | activate blood circulation, urinary and bowel obstruction, abortion, contraception, witchcraft poison antidote |  |  |  |
| *Iris* sp. |  | Iridaceae | herb | wild | tuber | decoct in water and take orally | hepatitis B, blood vessel health, bone setting | Guanxi | 0.0278 | HRC434 |
| *Iris tectorum* Maximowicz |  | Iridaceae | herb | wild | tuber | soak in wine and take internally | traumatic injury, activate blood circulation | Guanxi | 0.0556 | HRC285 |
| *Isotrema kwangsiense* (Chun & F. C. How ex C. F. Liang) X. X. Zhu, S. Liao & J. S. Ma | bai zhong guan | Aristolochiaceae | liana | wild or cultivated | tuber | decoct in water and take orally | anti-inflammatory | Yaoli | 0.0556 | HRC29 |
| *Ixeridium dentatum* (Thunberg) Tzvelev |  | Asteraceae | herb | wild | whole plant | decoct in water and take orally | high blood pressure | Yaoli, Yaozhai | 0.0741 | HRC119 |
| *Jasminum lanceolaria* Roxburgh |  | Oleaceae | liana | wild | stem | decoct in water and take orally | joint pain, joint loosening | Lile, Guanxi, Yaozhai | 0.1667 | HRC158 |
|  |  |  |  |  |  | decoct in water and wash externally | gynecological disorders |  |  |  |
| *Jasminum sinense* Hemsl. |  | Oleaceae | liana | wild | whole plant | decoct in water and wash externally | unblocking channels, paralysis | Dongjia | 0.0463 | HRC892 |
| *Jasminum* sp. |  | Oleaceae | liana | wild | whole plant | decoct in water and wash externally | traumatic injury | Huaili | 0.0370 | HRC636 |
| *Juglans regia* Linnaeus |  | Juglandaceae | tree | cultivated | leaf | decoct in water and wash externally | anti-itch | Guanxi | 0.0463 | HRC701 |
| *Justicia procumbens* Linnaeus |  | Acanthaceae | herb | wild | whole plant | take orally directly | throat obstruction | Yaoli, Yaozhai, Guanxi, Lile | 0.1389 | HRC23 |
|  |  |  |  |  |  | decoct in water and take orally | stones |  |  |  |
| *Kadsura coccinea* (Lemaire) A. C. Smith | ya gua | Schisandraceae | liana | wild | stem | soak in wine and take internally | traumatic injury, internal injury, rheumatism | Yaoshan, Lihu, Yaoli, Guanxi, Yaozhai, Lile, Baha | 0.2870 | HRC309 |
| *Kadsura longipedunculata* Finet & Gagnepain | pa teng | Schisandraceae | liana | wild | stem, root | decoct in water and take orally | internal injury | Yaoshan, Lihu, Yaoli, Guanxi, Yaozhai, Lile, Baha | 0.3426 | HRC8 |
|  |  |  |  |  |  | soak in wine and take internally | body strengthening, rheumatism, activate blood circulation and remove stasis |  |  |  |
|  |  |  |  |  |  | decoct in water and wash externally | traumatic injury |  |  |  |
| *Kalopanax septemlobus* (Thunberg) Koidzumi |  | Araliaceae | tree | wild | stem bark | soak in wine and take internally | rheumatism | Yaozhai | 0.0556 | HRC192 |
| *Kummerowia striata* (Thunberg) Schindler | e ke mang ka | Fabaceae | herb | wild | whole plant | decoct in water and take orally | pneumonia, gastritis, bone hyperplasia, traumatic injury | Yaoli, Yaoshan, Guanxi | 0.1389 | HRC6 |
|  |  |  |  |  |  | crush, extract juice, and take orally | abdominal pain and diarrhea |  |  |  |
|  |  |  |  |  |  | stew with pork liver | night blindness |  |  |  |
| *Leonurus japonicus* Houttuyn | zu ei gai min | Lamiaceae | herb | wild | whole plant | decoct in water and take orally | menstrual irregularities, infertility | Guanxi | 0.0741 | HRC364 |
| *Lepidogrammitis diversa* (Rosenst.) Ching |  | Polypodiaceae | herb | wild | leaf | soak in wine and apply externally | skin diseases | Lile | 0.0741 | HRC152 |
| *Lespedeza bicolor* Turczaninow |  | Fabaceae | shrub | wild | leaf | take orally directly | stomach pain | Yaozhai | 0.0556 | HRC338 |
|  |  |  |  |  |  | crush and apply topically | traumatic injury |  |  |  |
| *Lespedeza cuneata* (Dumont de Courset) G. Don | ye guan men | Fabaceae | shrub | wild | whole plant | decoct in water and take orally | diuretic, kidney nourishment | Yaozhai, Guanxi, Lihu | 0.2870 | HRC270 |
| *Ligularia hodgsonii* J. D. Hooker | zuo gou | Asteraceae | herb | wild | whole plant | decoct in water and take orally | consumptive lung disease, cough, tuberculosis | Guanxi, Yaozhai | 0.0926 | HRC310 |
| *Ligustrum lucidum* W. T. Aiton | tong yang | Oleaceae | tree | cultivated | fruit | decoct in water and wash externally | scald | Yaoshan | 0.0741 | HRC1422 |
|  |  |  |  |  | leaf | decoct in water and take orally | physical weakness, diarrhea, sore throat |  |  |  |
| *Ligustrum sinense* Loureiro |  | Oleaceae | shrub | wild | leaf | chew and apply topically | angular cheilitis | Yaozhai | 0.0370 | HRC324 |
| *Lilium brownii* var. *viridulum* Baker | gei | Liliaceae | herb | wild | corm | decoct in water and take orally | tuberculosis with coughing blood, cough with thick sputum, dry cough without phlegm | Lile, Yaozhai, Yaoshan | 0.0741 | HRC1465 |
|  |  |  |  |  |  | soak in wine and take internally | insomnia, irritability |  |  |  |
| *Lilium rosthornii* Diels | shan bai he | Liliaceae | herb | wild | corm | crush and apply topically | unidentified swelling and infection | Lile | 0.0185 | HRC247 |
| *Lindernia crustacea* (Linnaeus) F. Mueller |  | Linderniaceae | herb | wild | whole plant | crush and apply topically | snake bite | Guanxi | 0.0741 | HRC301 |
| *Lindernia setulosa* (Maximowicz) Tuyama ex H. Hara |  | Linderniaceae | herb | wild | whole plant | crush and apply topically | snake bite | Guanxi | 0.0556 | HRC675 |
| *Liquidambar formosana* Hance |  | Altingiaceae | tree | wild | tender leaf | take orally directly | stomach pain, hemiplegia | Lile, Huaili, Guanxi, Yaozhai, Yaoshan | 0.2778 | HRC127 |
| *Liriope spicata* (Thunberg) Loureiro | zhu shu | Asparagaceae | herb | wild | whole plant | decoct in water and take orally | rabid dog bite, back pain | Huaili, Guanxi, Baha | 0.2593 | HRC382 |
|  |  |  |  |  |  | soak in wine and take internally | witchcraft poison antidote |  |  |  |
| *Litsea cubeba* (Loureiro) Persoon |  | Lauraceae | tree | wild | root, fruit | soak in wine and take internally | cooling, pain relief | Yaoli | 0.0741 | HRC58 |
| *Lobelia chinensis* Loureiro | e chang dou weng | Campanulaceae | herb | wild | whole plant | crush and apply topically | snake bite poisoning, unidentified swelling and infection, furuncle | Yaozhai, Guanxi, Yaoshan | 0.1852 | HRC1441 |
|  |  |  |  |  |  | decoct in water and take orally | mastitis, edema |  |  |  |
|  |  |  |  |  |  | stew with meat | pediatric indigestion, dysentery |  |  |  |
| *Lobelia nummularia* Lamarck |  | Campanulaceae | herb | wild | whole plant | decoct in water and take orally | stones | Guanxi, Yaozhai | 0.0833 | HRC371 |
|  |  |  |  |  |  | crush and apply topically | shingles, witchcraft poison antidote |  |  |  |
| *Lonicera hypoglauca* Miquel |  | Caprifoliaceae | liana | wild | flower | decoct in water and take orally | cold and cough | Yaoshan, Dongjia | 0.1111 | HRC543 |
| *Lophatherum gracile* Brongniart in Duperrey | long tao li | Poaceae | herb | wild | whole plant | decoct in water and take orally | painful urination, blood in urine, mouth and tongue ulcers, red and swollen eyes | Yaoshan, Guanxi, Yaozhai | 0.1667 | HRC1455 |
| *Luffa aegyptiaca* Miller |  | Cucurbitaceae | liana | cultivated | fruit | crush and apply topically | mastitis | Lile | 0.0185 | HRC128 |
| *Lycianthes biflora* (Loureiro) Bitter | zhu bu shai | Solanaceae | herb | wild | whole plant | decoct in water and take orally | pesticide poisoning treatment | Guanxi | 0.0278 | HRC298 |
|  |  |  |  |  |  | soak in wine and take internally | blood tonic |  |  |  |
| *Lycium chinense* Miller | gu yi lai | Solanaceae | shrub | cultivated | tender bud | decoct in water and take orally | deficient heat with night sweating, clear heat and reduce fire, tuberculosis cough | Yaoshan, Huaili, Yaozhai | 0.1481 | HRC1430 |
|  |  |  |  |  | root bark | decoct in water and take orally | night sweats, tuberculosis cough, night sweating |  |  |  |
|  |  |  |  |  | fruit, leaf | stew with meat | back pain, physical weakness, dizziness |  |  |  |
| *Solanum lycopersicum* L. | bi gua | Solanaceae | herb | cultivated | leaf | crush and apply topically | burn and scald, snake bite | Guanxi, Lile | 0.0556 | HRC713 |
| *Lycoris radiata* (L’Heritier) Herbert | zhuo na ya hou | Amaryllidaceae | herb | wild | corm | decoct in water and take orally | activate blood circulation and remove stasis, witchcraft poison antidote | Guanxi, Yaozhai | 0.1389 | HRC314 |
|  |  |  |  |  |  | crush and apply topically | psoriasis |  |  |  |
| *Lygodium flexuosum* (L.) Sw. |  | Lygodiaceae | liana | wild | whole plant | decoct in water and take orally / decoct in water and wash externally | gynecological disorders | Yaoli | 0.0463 | HRC26 |
| *Lygodium japonicum* (Thunb.) Sw. | guo de gei | Lygodiaceae | liana | wild | whole plant | decoct in water and take orally | urinary tract infection, stones, hemiplegia | Yaoli, Guanxi, Yaozhai | 0.1019 | HRC78 |
|  |  |  |  |  |  | decoct in water and wash externally | joint pain, fatigue relief |  |  |  |
|  |  |  |  |  |  | use as bedding for sleeping | alleviate fatigue in the elderly |  |  |  |
| *Lysimachia christiniae* Hance |  | Primulaceae | herb | wild | whole plant | decoct in water and take orally | stomach pain | Lile, Yaozhai | 0.0556 | HRC134 |
| *Lysimachia fortunei* Maximowicz | e long pi zha | Primulaceae | herb | wild | root | decoct in water and take orally | jaundice, hepatitis | Yaoli, Guanxi | 0.0833 | HRC41 |
|  |  |  |  |  |  | crush and apply topically | traumatic injury, hemostasis, remove necrotic tissue and promote granulation |  |  |  |
| *Lysimachia tianyangensis* D. Fang & C. Z. Gao in C. Z. Gao |  | Primulaceae | herb | wild | whole plant | decoct in water and take orally | liver cirrhosis | Guanxi | 0.0185 | HRC393 |
| *Lysionotus pauciflorus* Maximowicz | dao wei ai | Gesneriaceae | herb | wild | whole plant | decoct in water and take orally | witchcraft poison antidote, regulate menstruation, relieve cough and expectoration, toothache | Lile, Dongjia, Yaozhai, Guanxi, Yaoshan, Baha | 0.1759 | HRC566 |
|  |  |  |  |  |  | crush and apply topically | skin itching |  |  |  |
|  |  |  |  |  |  | decoct in water and wash externally | physical weakness |  |  |  |
| *Maclura cochinchinensis* (Loureiro) Corner | li bu ru | Moraceae | shrub | wild | root | soak in wine and take internally | traumatic injury, seminal conservation | Yaozhai, Yaoli | 0.1111 | HRC68 |
|  |  |  |  |  |  | decoct in water and take orally | tuberculosis, kidney stones |  |  |  |
| *Mahonia bealei* (Fortune) Carriere |  | Berberidaceae | shrub | wild | old stem | decoct in water and take orally | anti-inflammatory, stomach pain | Guanxi | 0.0556 | HRC399 |
| *Mahonia fortunei* (Lindley) Fedde | tong ke li | Berberidaceae | shrub | wild | stem | decoct in water and take orally | pneumonia, jaundice hepatitis, anti-inflammatory, stomach pain | Yaoli | 0.0648 | HRC73 |
| *Mallotus philippensis* (Lamarck) Muller Argoviensis |  | Euphorbiaceae | shrub | wild | leaf | crush and apply topically | scar removal and skin regeneration | Guanxi | 0.0278 | HRC282 |
|  |  |  |  |  |  | decoct in water and take orally | jaundice hepatitis |  |  |  |
| *Mallotus repandus* (Willdenow) Muller Argoviensis | zhuo na ya hou | Euphorbiaceae | shrub | wild | leaf | crush and apply topically | remove necrotic tissue and promote granulation, traumatic injury | Dongjia, Guanxi, Baha | 0.0833 | HRC431 |
|  |  |  |  |  |  | soak in wine and take internally | joint pain |  |  |  |
|  |  |  |  |  |  | decoct in water and wash externally | unblocking channels, paralysis |  |  |  |
| *Marchantia polymorpha* L. |  | Marchantiaceae | herb | wild | whole plant | crush and apply topically | onychomycosis | Lile, Guanxi | 0.0278 | HRC678 |
| *Melastoma dodecandrum* Loureiro | mo weng xiang | Melastomataceae | herb | wild | leaf | crush and apply topically | anti-itch | Yaoshan | 0.0556 | HRC445 |
| *Melastoma malabathricum* Linnaeus | e ka chao la | Melastomataceae | shrub | wild | root | crush and apply topically | traumatic injury | Yaoli, Lihu, Yaozhai | 0.1481 | HRC493 |
|  |  |  |  |  |  | Sun-dry, grind and apply externally | external injury bleeding |  |  |  |
|  |  |  |  |  |  | decoct in water and take orally | menorrhagia, menstrual irregularities, dysentery, joint swelling and pain |  |  |  |
|  |  |  |  |  |  | steam with meat | postpartum low milk supply |  |  |  |
| *Melia azedarach* Linnaeus |  | Meliaceae | tree | wild or cultivated | root | decoct in water and take orally | skin carbuncles | Lile | 0.0648 | HRC210 |
| *Mentha canadensis* Linnaeus | yang gei nie | Lamiaceae | herb | cultivated | whole plant | chew and apply topically | gum swelling and pain | Guanxi, Yaozhai, Huaili | 0.1944 | HRC312 |
| *Microsorum fortunei* (T. Moore) Ching | ya bia fu | Polypodiaceae | herb | wild | whole plant | decoct in water and take orally | stones, stomach pain, gastritis | Guanxi | 0.0463 | HRC436 |
| *Miliusa sinensis* Finet & Gagnepain |  | Annonaceae | shrub | wild | stem | soak in wine and take internally | shingles | Lile | 0.0278 | HRC154 |
| *Miscanthus sinensis* Andersson | gu yu | Poaceae | herb | wild | root, stem | decoct in water and take orally | painful urination, cough | Yaoshan | 0.0370 | HRC1456 |
| *Mollugo stricta* Linnaeus |  | Molluginaceae | herb | wild | whole plant | chew and ingest | stomach pain, indigestion | Dongjia | 0.0463 | HRC263 |
| *Morus alba* Linnaeus | e ji | Moraceae | tree | cultivated | root | decoct in water and take orally | deworming, cough | Yaoshan, Yaozhai, Lile | 0.0833 | HRC108 |
|  |  |  |  |  |  | crush and apply topically | bone setting |  |  |  |
| *Mucuna birdwoodiana* Tutcher |  | Fabaceae | liana | wild | stem, root | decoct in water and wash externally | paralysis, traumatic injury | Huaili, Lihu, Yaoshan, Guanxi, Yaozhai, Yaoli, Lile | 0.4259 | HRC306 |
|  |  |  |  |  |  | soak in wine and take internally / decoct in water and take orally | blood tonic, joint pain |  |  |  |
| *Murdannia triquetra* (Wallich ex C. B. Clarke) Bruckner in Engler & Prantl |  | Commelinaceae | herb | wild | whole plant | decoct in water and take orally | diuretic | Yaoli | 0.0556 | HRC82 |
| *Murraya euchrestifolia* Hayata |  | Rutaceae | shrub | wild | branches and leaves | decoct in water and take orally | cough | Lile | 0.0741 | HRC524 |
| Murraya exotica L. Mant. |  | Rutaceae | shrub | wild | whole plant | decoct in water and wash externally | hemiplegia, joint pain, gout | Lile | 0.0556 | HRC234 |
| *Musa basjoo* Siebold & Zuccarini |  | Musaceae | herb | cultivated | inflorescence | steam with meat | stomach pain, gastritis | Baha | 0.0463 | HRC554 |
| *Musella lasiocarpa* (Franchet) C. Y. Wu ex H. W. Li |  | Musaceae | herb | cultivated | leaf | crush and apply topically | abdominal bloating | Yaozhai | 0.0278 | HRC468 |
| *Mussaenda pubescens* W. T. Aiton | e bei liang | Rubiaceae | shrub | wild | whole plant | decoct in water and take orally | external heat causing fever, child food stagnation fever, short and red urine, uterine bleeding | Yaoli, Yaozhai, Yaoshan | 0.1759 | HRC1434 |
| *Morella rubra* Lour. | di ma | Myricaceae | tree | wild | bark | soak in water and gargle | gum swelling and pain | Guanxi, Yaozhai | 0.1667 | HRC651 |
|  |  |  |  |  |  | soak in wine and wash externally | syphilis |  |  |  |
| *Nandina domestica* Thunberg | tong jiao ke | Berberidaceae | shrub | wild | stem, root | soak in wine and take internally | back pain, stomach pain, cold, cough | Yaoli, Huaili, Guanxi, Yaoshan | 0.3519 | HRC90 |
|  |  |  |  |  |  | decoct in water and take orally | witchcraft poison antidote, tuberculosis |  |  |  |
| *Nanhaia speciosa* (Champ. ex Benth.) J. Compton & Schrire | mo gu piao la | Fabaceae | herb | wild or cultivated | root | soak in wine and take internally | joint pain, fatigue relief | Lile, Yaoshan, Guanxi | 0.2593 | HRC358 |
| *Nekemias cantoniensis* (Hook. & Arn.) J. Wen & Z. L. Nie | pi kua nong | Vitaceae | liana | wild | branches and leaves | decoct in water and take orally | joint pain | Guanxi, Lile | 0.1111 | HRC704 |
| *Neocinnamomum lecomtei* H. Liu |  | Lauraceae | tree | wild | stem, root | soak in wine and take internally / soak in wine and apply externally | pain relief | Lile | 0.0185 | HRC151 |
| *Neottopteris antrophyoides* (Christ) Ching |  | Aspleniaceae | herb | wild | leaf | crush and apply topically | shingles | Lile | 0.0185 | HRC150 |
| *Nephrolepis cordifolia* (L.) C. Presl |  | Nephrolepidaceae | herb | wild | bulb | decoct in water and take orally | child cough, fever | Yaoli, Lile, Guanxi, Baha | 0.4630 | HRC197 |
|  |  |  |  |  |  | crush and apply topically | knife wound, external injury, hernia |  |  |  |
|  |  |  |  |  |  | take orally directly | male genital swelling, dysentery, diuretic |  |  |  |
| *Odontosoria chinensis* J. Sm. | kuo gu cao | Lindsaeaceae | herb | wild | tender leaf | crush and apply topically | tumor, abscess | Yaoli | 0.0833 | HRC12 |
|  |  |  |  |  |  | decoct in water and take orally | detoxification, unidentified swelling and infection, joint dislocation |  |  |  |
| *Oenothera rosea* L’Heritier ex Aiton | ya ai | Onagraceae | herb | wild | whole plant | decoct in water and take orally | anti-inflammatory | Guanxi | 0.0741 | HRC404 |
| *Ohwia caudata* (Thunberg) H. Ohashi |  | Leguminosae | shrub | wild | whole plant | crush and apply topically | unidentified swelling and infection, joint dislocation | Yaoli, Lile, Guanxi | 0.2037 | HRC39 |
| *Onychium japonicum* (Thunb.) Kunze |  | Pteridaceae | herb | wild | whole plant | decoct in water and wash externally | unidentified swelling and infection | Dongjia, Guanxi, Yaoli | 0.0556 | HRC897 |
| *Ophioglossum vulgatum* L. | e jiu qi | Ophioglossaceae | herb | wild | whole plant | decoct in water and take orally | abdominal pain, breast pain | Guanxi, Yaoshan | 0.0556 | HRC1184 |
|  |  |  |  |  |  | crush and apply topically | snake bite |  |  |  |
| *Opuntia dillenii* (Ker Gawler) Haworth |  | Cactaceae | herb | wild | leaf | crush and apply topically | anti-inflammatory, infection swelling | Yaozhai, Guanxi, Yaoshan, Huaili | 0.2407 | HRC325 |
|  |  |  |  |  |  | steam with meat | shingles |  |  |  |
| *Oreocnide frutescens* (Thunberg) Miquel |  | Urticaceae | shrub | wild | branches and leaves | decoct and hold in mouth | gum swelling and pain | Dongjia | 0.0556 | HRC908 |
| *Osbeckia stellata* Ham. ex D. Don: C. B. Clarke | ge ka qi ke | Melastomataceae | shrub | wild | whole plant | decoct in water and take orally | dysentery, bloody dysentery, amenorrhea, cough | Guanxi, Yaoli | 0.1204 | HRC288 |
| *Oxalis corniculata* Linnaeus | jia | Oxalidaceae | herb | wild | whole plant | crush and apply topically | traumatic injury, scald, toothache | Guanxi, Yaoshan | 0.1019 | HRC1221 |
|  |  |  |  |  |  | decoct in water and take orally | mumps, sore throat, jaundice, insomnia |  |  |  |
| *Oxyspora paniculata* (D. Don) Candolle | li hong | Melastomataceae | shrub | wild | branches and leaves | decoct in water and take orally | prostatitis, painful urination | Yaoli | 0.0463 | HRC45 |
| *Paederia foetida* Linnaeus | ge ba | Rubiaceae | liana | wild | tender leaf | crush and apply topically | corns, calluses, non-healing ulcers, neurodermatitis | Yaoli, Guanxi, Lile, Yaozhai, Baha, Lihu | 0.2685 | HRC15 |
|  |  |  |  |  |  | decoct in water and take orally | organophosphate pesticide poisoning |  |  |  |
|  |  |  |  |  |  | decoct, mix with alcohol, and take orally | traumatic injury |  |  |  |
|  |  |  |  |  |  | stew with meat | stomach gas pain, pediatric indigestion |  |  |  |
| *Paederia pertomentosa* Merrill ex H. L. Li | chou teng | Rubiaceae | liana | wild | root | decoct in water and take orally | jaundice, hepatitis, infectious diseases | Yaoli | 0.0556 | HRC63 |
| *Palhinhaea cernua* (L.) Franco et Vasc. |  | Lycopodiaceae | herb | wild | whole plant | decoct in water and wash externally | unblocking meridians | Yaoli, Guanxi, Lihu, Yaoshan | 0.2407 | HRC102 |
| *Paphiopedilum micranthum* Tang & F. T. Wang |  | Orchidaceae | herb | wild | whole plant | decoct in water and take orally | diabetes | Yaoli | 0.0185 | HRC4 |
| *Paris cronquistii* (Takhtajan) H. Li |  | Melanthiaceae | herb | wild | tuber, leaf | sun-dry, grind, and apply externally. | remove necrotic tissue and promote granulation | Yaoli | 0.0463 | HRC624 |
|  |  |  |  |  |  | crush and apply topically | snake bite, traumatic injury |  |  |  |
| *Paris fargesii* Franchet |  | Melanthiaceae | herb | wild or cultivated | tuber | crush and apply topically | snake bite | Guanxi | 0.0556 | HRC455 |
|  |  |  |  |  |  | soak in water and take orally / soak in wine and take internally | cancer |  |  |  |
| *Paris polyphylla* Smith in Rees | e tuo cai | Melanthiaceae | herb | wild or cultivated | tuber | crush and apply topically | snake bite, unidentified swelling and infection | Yaoshan | 0.0463 | HRC1461 |
|  |  |  |  |  |  | decoct in water and take orally | stomach heat pain |  |  |  |
|  |  |  |  |  |  | sun-dry, grind, mix with water, and take orally | insufficient lactation in women |  |  |  |
| *Parthenocissus semicordata* (Wallich) Planchon in A. Candolle & C. Candolle | hie yang | Vitaceae | liana | wild | whole plant | decoct in water and wash externally | body weakness and soreness, fatigue relief | Yaozhai | 0.0278 | HRC655 |
| *Parthenocissus suberosa* Handel-Mazzetti | shi tao teng | Vitaceae | liana | wild | whole plant | decoct in water and take orally / decoct in water and wash externally | gynecological disorders (postpartum diseases) | Yaoli | 0.0278 | HRC65 |
| *Parthenocissus tricuspidata* (Siebold & Zuccarini) Planchon in A. Candolle & C. Candolle | ya pa feng | Vitaceae | herb | wild | whole plant | crush and apply topically | traumatic injury, bone setting | Baha | 0.0185 | HRC346 |
| *Passiflora cupiformis* Masters |  | Passifloraceae | liana | wild | whole plant | decoct in water and take orally | unidentified swelling and pain, pharyngitis, traumatic injury, joint pain | Yaozhai, Baha | 0.0463 | HRC175 |
| *Patrinia scabiosifolia* Link |  | Caprifoliaceae | herb | wild | whole plant | decoct in water and wash externally | hemiplegia | Lile | 0.0463 | HRC231 |
| *Patrinia villosa* (Thunb.) Juss. | e hai | Caprifoliaceae | herb | wild | whole plant, leaf | crush and apply topically | unidentified swelling and infection, mumps | Yaoshan | 0.0556 | HRC1439 |
|  |  |  |  |  |  | decoct in water and take orally | dysentery, sore throat |  |  |  |
| *Perilla frutescens* (Linnaeus) Britton | e liao yi | Lamiaceae | herb | cultivated | leaf | decoct in water and take orally / steam with eggs | bloody dysentery, diarrhea | Yaozhai, Guanxi, Huaili | 0.1944 | HRC585 |
| *Perilla frutescens* var. *purpurascens* (Hayata) H. W. Li |  | Lamiaceae | herb | wild | branches and leaves | decoct in water and take orally | cerebral hyperemia, postpartum bleeding | Dongjia, Baha | 0.1481 | HRC536 |
|  |  |  |  |  |  | crush and insert into nostrils | nosebleed |  |  |  |
|  |  |  |  |  |  | decoct in water and wash externally | unblocking channels, paralysis |  |  |  |
| *Peristrophe japonica* (Thunberg) Bremekamp |  | Acanthaceae | herb | wild | whole plant | crush and apply topically | traumatic injury | Guanxi, Yaozhai | 0.1019 | HRC299 |
|  |  |  |  |  |  | take orally directly | antidote for poisoning, throat obstruction, witchcraft poison antidote |  |  |  |
|  |  |  |  |  |  | soak in wine and take internally / decoct in water and take orally | abdominal pain |  |  |  |
| *Persicaria capitata* (Buch.-Ham. ex D. Don) H. Gross |  | Polygonaceae | herb | wild | whole plant | crush and apply topically | hemorrhoids, skin itching | Lile, Guanxi, Huaili, Yaoshan | 0.2778 | HRC201 |
|  |  |  |  |  |  | take orally directly | abdominal pain |  |  |  |
|  |  |  |  |  |  | decoct in water and wash externally | gynecological disorders, syphilis |  |  |  |
| *Persicaria filiformis* (Thunb.) Nakai |  | Polygonaceae | herb | wild | tuber | soak in wine and take internally | blood tonic | Guanxi | 0.0556 | HRC449 |
| *Peucedanum guangxiense* R. H. Shan & M. L. Sheh | ba cuo | Apiaceae | herb | wild | whole plant | decoct in water and take orally / soak in wine and take internally | headache due to wind, blood circulation | Yaozhai, Guanxi | 0.0741 | HRC169 |
| *Phaius columnaris* C. Z. Tang & S. J. Cheng |  | Orchidaceae | herb | wild | corm | soak in wine and take internally | erectile dysfunction, penile shrinkage | Lile, Guanxi | 0.1759 | HRC156 |
| *Phanera aurea* (H. Lév.) Mackinder & R. Clark |  | Fabaceae | liana | wild | branches and leaves | decoct in water and take orally | diuretic | Guanxi, Lile | 0.0741 | HRC380 |
|  |  |  |  |  |  | soak in wine and take internally | urinary obstruction, kidney nourishment |  |  |  |
| *Phedimus aizoon* (Linnaeus) 't Hart |  | Crassulaceae | herb | cultivated | leaf | crush and apply topically | scald | Yaoshan | 0.0926 | HRC503 |
| *Pholidota yunnanensis* Rolfe | zu nie mie | Orchidaceae | herb | wild | whole plant | decoct in water and take orally | tuberculosis, liver cirrhosis | Guanxi | 0.0833 | HRC265 |
| *Phragmites australis* (Cavanilles) Trinius ex Steudel | yi guo | Poaceae | herb | cultivated | stem | decoct in water and take orally | rabid dog bite | Guanxi, Yaozhai | 0.0556 | HRC383 |
| *Phyllostachys nigra* (Loddiges ex Lindley) Munro | zhou su | Poaceae | herb | wild | stem | decoct in water and take orally | rabid dog bite | Guanxi | 0.0278 | HRC384 |
| *Physalis angulata* L. | wo za | Solanaceae | herb | wild | whole plant | crush and apply topically | pediatric hernia | Lile, Guanxi | 0.1111 | HRC649 |
| *Phytolacca acinosa* Roxburgh |  | Phytolaccaceae | herb | wild | root | stew with meat | immune boosting | Yaozhai, Guanxi, Yaoli, Lihu | 0.0833 | HRC417 |
| *Phytolacca americana* Linnaeus | ya lai guo | Phytolaccaceae | herb | wild | fruit | soak in wine and take internally | blood tonic | Yaozhai, Guanxi, Yaoli, Lihu | 0.3889 | HRC682 |
|  |  |  |  |  | tuber | stew with meat | immune boosting |  |  |  |
| *Pinellia cordata* N. E. Brown |  | Araceae | herb | wild | tuber | soak in wine and take internally / soak in wine and apply externally | snake bite | Yaozhai, Guanxi, Lile | 0.1111 | HRC258 |
| *Pinellia ternata* (Thunberg) Tenore ex Breitenbach | ban bu tiao | Araceae | herb | wild | tuber | crush and apply topically | corns, calluses | Yaoli, Guanxi, Lile | 0.0741 | HRC14 |
| *Pittosporum tonkinense* Gagnepain |  | Pittosporaceae | shrub | wild | branches and leaves | decoct in water and take orally | high blood pressure | Guanxi, Yaoshan | 0.0278 | HRC279 |
| *Plantago asiatica* Linnaeus | e tou mu | Plantaginaceae | herb | wild | whole plant | decoct in water and take orally | urinary tract infection, jaundice, hepatitis, fever reduction, stones, dysentery, indigestion | Yaoli, Guanxi, Yaozhai, Lihu, Huaili, Yaoshan | 0.3056 | HRC84 |
| *Platycarya strobilacea* Siebold & Zuccarini |  | Juglandaceae | tree | wild | branchlet | decoct in water and wash externally | skin itching | Guanxi | 0.0278 | HRC177 |
| *Platycladus orientalis* (Linnaeus) Franco | long | Cupressaceae | tree | cultivated | branches and leaves, seed | decoct in water and take orally | bloody stool, nosebleed, heavy menstruation, constipation, hair loss | Guanxi, Yaoshan | 0.0833 | HRC1186 |
|  |  |  |  |  | branches and leaves | crush and apply topically | external injury bleeding |  |  |  |
| *Platycodon grandiflorus* (Jacquin) A. Candolle | e hao ka | Campanulaceae | herb | wild | root | decoct in water and take orally | tuberculosis, sore throat, cough with thick sputum | Yaoshan | 0.0185 | HRC1442 |
| *Pleuropterus multiflorus* (Thunb.) Nakai | ren dou zhuai | Polygonaceae | liana | wild | tuber | soak in wine and take internally | immune boosting, aid digestion | Yaoli, Yaoshan, Guanxi, Yaozhai, Lile, Lihu, Dongjia | 0.1019 | HRC9 |
|  |  |  |  |  |  | stew with meat | kidney deficiency with nocturnal emission, blood deficiency with dizziness, premature greying, physical weakness |  |  |  |
| *Polygala japonica* Houttuyn | jie ti qiao | Polygalaceae | herb | wild | whole plant | crush and apply topically | cataract | Huaili, Yaoshan, Guanxi | 0.1111 | HRC603 |
|  |  |  |  |  |  | decoct in water and take orally | diuretic |  |  |  |
|  |  |  |  |  |  | steam with pork liver | insomnia |  |  |  |
| *Polygonatum cyrtonema* Hua | zu suo xi | Asparagaceae | herb | wild | tuber | stew with meat | immune boosting | Lile, Huaili, Guanxi, Yaozhai, Baha, Yaoshan | 0.3889 | HRC194 |
| *Polygonatum kingianum* Collett & Hemsley |  | Asparagaceae | herb | wild | tuber | crush, mix with water, and take orally | abortion, contraception | Baha, Yaozhai, Lile, Guanxi, Huaili, Lihu, Yaoli, Yaoshan | 0.2778 | HRC475 |
|  |  |  |  |  |  | stew with meat | immune boosting |  |  |  |
| *Polygonum aviculare* Linnaeus | tong ya mi | Polygonaceae | herb | wild | whole plant | decoct in water and take orally | painful urination, dysentery | Guanxi, Yaoshan | 0.0556 | HRC1191 |
|  |  |  |  |  |  | decoct in water for external wash | female genital itching, anal itching |  |  |  |
| *Polygonum chinense* Linnaeus | e la lv | Polygonaceae | herb | wild | whole plant | decoct in water and take orally | diarrhea, dysentery, vaginal discharge, jaundice | Lile, Yaoshan, Guanxi, Yaoshan | 0.3333 | HRC1195 |
|  |  |  |  |  |  | make soup | traumatic injury, high blood pressure |  |  |  |
| *Polygonum odoratum* Lour. | e liao you | Polygonaceae | herb | wild | whole plant | decoct in water and take orally | diarrhea, dysentery, pediatric indigestion | Guanxi | 0.0741 | HRC280 |
|  |  |  |  |  |  | crush and apply topically | athlete's foot |  |  |  |
|  |  |  |  |  |  | decoct in water for external wash | traumatic injury, rheumatic bone pain |  |  |  |
| *Polygonum perfoliatum* Linnaeus | nuo ba da | Polygonaceae | herb | wild | whole plant | crush and apply topically | gynecological disorders, hemorrhoids, snake bite | Yaozhai, Lile, Guanxi | 0.1667 | HRC368 |
|  |  |  |  |  |  | decoct in water and take orally | pediatric fever with convulsions |  |  |  |
|  |  |  |  |  |  | decoct in water for external wash | shingles, eczema, cough |  |  |  |
| *Polygonum runcinatum* var. *sinense* Hemsley | jio bao feng | Polygonaceae | herb | wild | whole plant | crush and apply topically | traumatic injury, bone setting, snake bite | Guanxi, Yaozhai, Yaoli, Baha | 0.3796 | HRC330 |
|  |  |  |  |  |  | cook with chicken | immune boosting |  |  |  |
|  |  |  |  |  |  | decoct in water and take orally | infertility |  |  |  |
| *Portulaca oleracea* Linnaeus | e ni mei sha hong | Portulacaceae | herb | wild | whole plant | decoct in water and take orally | dysentery, herbal remedy for dysentery, pediatric indigestion, menorrhagia, acute appendicitis | Yaoshan, Huaili, Yaozhai | 0.1667 | HRC1196 |
|  |  |  |  |  |  | crush and apply topically | insect bite poisoning, unidentified swelling and infection, shingles |  |  |  |
|  |  |  |  |  |  | make soup | summer heatstroke with vomiting and diarrhea, coughing blood |  |  |  |
| *Potentilla freyniana* Bornmuller | jiu duo me | Rosaceae | herb | wild | root | decoct in water and take orally | diarrhea, dysentery | Yaoshan | 0.0556 | HRC1212 |
|  |  |  |  |  |  | cush, mix with alcohol, and take orally | traumatic injury |  |  |  |
|  |  |  |  |  |  | stew with meat | stomach pain, postpartum weakness in women |  |  |  |
|  |  |  |  |  | whole plant | crush and apply topically | snake bite |  |  |  |
| *Potentilla kleiniana* Wight & Arnott | ya pa ou | Rosaceae | herb | wild | whole plant | decoct in water and take orally | enteritis | Guanxi | 0.0741 | HRC296 |
| *Prunella vulgari*s Linnaeus | ya jing na | Lamiaceae | herb | wild | whole plant | crush and apply topically | hemorrhoids, lacquer allergy, hyperthyroidism, high blood pressure, dizziness, lymphadenopathy, painful urination, headache | Yaoshan, Huaili, Guanxi, Yaozhai | 0.2685 | HRC406 |
| *Pseudognaphalium affine* (D. Don) Anderberg | e ai dao | Asteraceae | herb | wild | whole plant | decoct in water and take orally | cold and cough, dizziness, blurred vision, high blood pressure | Yaozhai, Dongjia, Yaoshan | 0.1944 | HRC1447 |
|  |  |  |  |  |  | soak in wine and take internally | muscle and bone pain |  |  |  |
| *Pteris multifida* Poir. | e ai hao dao | Pteridaceae | herb | wild | whole plant | crush, mix with wine, and apply externally | centipede bite | Dongjia, Lile, Yaoshan | 0.1574 | HRC522 |
|  |  |  |  |  |  | crush and apply topically | tendon and bone setting, minor scald |  |  |  |
|  |  |  |  |  |  | decoct in water and take orally / soak in wine and take internally | traumatic injury, diarrhea, painful urination, jaundice hepatitis |  |  |  |
| *Pterolobium punctatum* Hemsley |  | Fabaceae | liana | wild | leaf | crush and apply topically | venomous snake bite, snake bite | Lile, Yaozhai, Guanxi | 0.1019 | HRC137 |
|  |  |  |  |  |  | decoct in water and wash externally | eye pain |  |  |  |
| *Pueraria montana* (Loureiro) Merrill | mo fei | Fabaceae | liana | wild | flower | soak in water and take orally | hangover relief | Guanxi, Yaoli, Huaili | 0.0741 | HRC167 |
|  |  |  |  |  |  | decoct in water and take orally | high blood pressure |  |  |  |
| *Pyracantha fortuneana* (Maximowicz) H. L. Li |  | Rosaceae | shrub | wild | root | decoct in water and take orally | dysentery | Lile | 0.0185 | HRC215 |
| *Pyrrosia lingua* (Thunb.) Farwell | e dan gao | Polypodiaceae | herb | wild | whole plant | decoct in water and take orally | stones | Yaozhai, Yaoshan | 0.1574 | HRC174 |
| *Pyrrosia tonkinensis* (Giesenh.) Ching |  | Polypodiaceae | herb | wild | whole plant | decoct in water and take orally | stones | Yaozhai, Guanxi | 0.0833 | HRC185 |
| *Radermachera hainanensis* Merrill | zhuo tou suo | Bignoniaceae | tree | wild | root | crush and apply topically | bone setting | Guanxi | 0.0185 | HRC295 |
| *Ranunculus chinensis* Bunge |  | Ranunculaceae | herb | wild | whole plant | crush, extract juice, and drip onto affected area | pterygium | Yaoli, Yaozhai | 0.0741 | HRC56 |
| *Raphanus sativus* Linnaeus | gu tou | Brassicaceae | herb | cultivated | seed | decoct in water and take orally | food stagnation, swelling reduction, constipation, high blood pressure, hyperlipidemia | Yaoshan | 0.0556 | HRC1209 |
|  |  |  |  |  | leaf | crush and apply topically | nosebleed |  |  |  |
|  |  |  |  |  | tuber | crush and apply topically | mouth ulcers |  |  |  |
| *Reineckea carnea* (Andrews) Kunth | e long wei rao | Asparagaceae | herb | wild or cultivated | whole plant | soak in wine and take internally / decoct in water and take orally | witchcraft poison antidote | Huaili, Lile | 0.1574 | HRC578 |
|  |  |  |  |  |  | crush, mix with alcohol, and apply externally | traumatic injury |  |  |  |
|  |  |  |  |  |  | stew with pork lungs | pediatric malnutrition and anorexia |  |  |  |
| *Reynoutria japonica* Houttuyn | a gua | Polygonaceae | herb | wild | root | grind and apply externally | traumatic injury, bone setting | Guanxi, Yaozhai, Yaoli, Lihu | 0.2315 | HRC618 |
|  |  |  |  |  |  | decoct in water and take orally | jaundice, pneumonia cough, edema, dysentery, lower blood lipids |  |  |  |
| *Rhamnus utilis* Decaisne |  | Rhamnaceae | shrub | wild | branches and leaves | decoct in water and take orally | rabies prevention | Yaoshan, Yaoli, Guanxi, Yaozhai | 0.3333 | HRC504 |
|  |  |  |  |  | root | decoct in water and wash externally | insecticide, anti-itch |  |  |  |
|  |  |  |  |  | fruit | crush and apply topically | gum inflammation |  |  |  |
| *Rhus chinensis* Mill. | bo pu | Anacardiaceae | shrub | wild | gall | decoct in water and wash externally | joint pain, fatigue relief | Baha, Yaozhai, Lile, Guanxi, Yaoshan | 0.3056 | HRC274 |
|  |  |  |  |  |  | crush and apply topically | bone setting |  |  |  |
|  |  |  |  |  |  | decoct in water and take orally | chronic bronchitis |  |  |  |
| *Ricinus communis* Linnaeus | de he pu | Euphorbiaceae | herb | wild | leaf | crush and apply topically | hemiplegia, prolapse | Guanxi, Yaozhai | 0.0741 | HRC422 |
| *Rohdea japonica* (Thunberg) Roth | jio bie nong | Asparagaceae | herb | wild | whole plant | chew and ingest | witchcraft poison antidote | Lihu, Yaozhai, Guanxi, Baha | 0.4167 | HRC162 |
|  |  |  |  |  |  | steam with eggs | angina |  |  |  |
|  |  |  |  |  |  | decoct in water and take orally | stomach pain |  |  |  |
| *Rosa chinensis* Jacquin |  | Rosaceae | shrub | cultivated | branches and leaves | decoct in water and take orally | menstrual irregularities, infertility | Guanxi | 0.0463 | HRC365 |
| *Rosa laevigata* Michaux | ye bu gei ka | Rosaceae | shrub | wild | root | decoct in water and take orally / soak in wine and take internally | aphrodisiac, seminal retention, menorrhagia, menstrual irregularities | Yaoli, Guanxi, Yaozhai, Lihu, Huaili, Yaoshan | 0.5370 | HRC46 |
|  |  |  |  |  |  | stew with meat | hemorrhoids |  |  |  |
|  |  |  |  |  |  | crush and apply topically | burn and scald, traumatic injury |  |  |  |
| *Rosa roxburghii* Trattinnick | la ke tong | Rosaceae | shrub | wild | root | decoct in water and take orally | abdominal bloating and loss of appetite, diarrhea | Yaoshan, Yaozhai | 0.0556 | HRC1213 |
|  |  |  |  |  | leaf | soak in wine and take internally | traumatic injury |  |  |  |
|  |  |  |  |  | fruit | decoct in water and take orally | poor appetite |  |  |  |
| *Rubia alata* Wallich in Roxburgh | si fang cao | Rubiaceae | herb | wild | whole plant | decoct in water and take orally / soak in wine and take internally | blood tonic, menstrual irregularities, amenorrhea, activate blood circulation and remove stasis, stones | Dongjia, Huaili, Yaozhai, Guanxi, Lile, Baha | 0.2778 | HRC178 |
|  |  |  |  |  | root | chew and hold in mouth | toothache |  |  |  |
| *Rubia cordifolia* Linnaeus | hei ga gou | Rubiaceae | liana | wild | whole plant | chew and hold in mouth | toothache | Yaoshan, Dongjia, Huaili, Yaozhai, Guanxi, Lile, Baha | 0.1019 | HRC652 |
|  |  |  |  |  |  | decoct in water and wash externally | joint pain |  |  |  |
| *Rubia wallichiana* Decaisne | si yao cao | Rubiaceae | liana | wild | root | crush, extract juice, and apply to affected area | foreign body in the eye, dental caries, gum swelling and pain | Yaoli | 0.0278 | HRC64 |
| *Rubus alceifolius* Poiret in Lamarck |  | Rosaceae | shrub | wild | root | decoct in water and take orally | menorrhagia, menstrual irregularities | Yaoshan, Yaozhai | 0.1019 | HRC497 |
| *Rubus lambertianus* Seringe in Candolle |  | Rosaceae | liana | wild | root | decoct in water and take orally | dysentery | Lile | 0.0278 | HRC214 |
| *Rubus niveus* Thunberg | wu pao ging | Rosaceae | shrub | wild | root | decoct in water and take orally | gastritis, cramps | Yaoli | 0.0278 | HRC7 |
| *Rubus parvifolius* Linnaeus | ging gei | Rosaceae | herb | wild | root | decoct in water and take orally | heavy menstruation, stomach pain, urinary stones | Yaoli | 0.0463 | HRC104 |
| *Rubus rosifolius* Smith | gei du | Rosaceae | herb | wild | whole plant | decoct in water and take orally | gum swelling and pain | Yaozhai, Huaili, Guanxi, Yaoli | 0.2593 | HRC484 |
|  |  |  |  |  |  | decoct and use for external wash | body itch |  |  |  |
| *Rumex nepalensis* Sprengel |  | Polygonaceae | herb | wild | tuber | take orally directly | sore throat | Huaili, Yaozhai | 0.1019 | HRC426 |
|  |  |  |  |  |  | crush and apply topically | mumps |  |  |  |
| *Sambucus javanica* Blume | hei jie feng | Adoxaceae | herb | wild | branches and leaves | decoct in water for external wash | anti-inflammatory, edema | Huaili, Yaoli, Guanxi, Yaozhai, Lile | 0.1759 | HRC631 |
|  |  |  |  |  |  | crush and apply topically | traumatic injury, remove stasis, bone setting |  |  |  |
| *Sambucus williamsii* Hance | tong tao tuo | Adoxaceae | shrub | cultivated | branches and leaves | crush and apply topically | bone setting | Guanxi | 0.0463 | HRC925 |
| *Sanicula chinensis* Bunge | e wen hua | Apiaceae | herb | wild | whole plant | decoct in water and take orally | dysmenorrhea | Huaili, Guanxi, Baha | 0.1019 | HRC1215 |
|  |  |  |  |  |  | stew with meat | dizziness, kidney deficiency causing dizziness, physical weakness with night sweats |  |  |  |
| *Sarcandra glabra* (Thunberg) Nakai |  | Chloranthaceae | shrub | wild | whole plant | decoct in water and take orally / soak in wine and take internally | traumatic injury, internal injury, periarthritis, abdominal pain, back pain, joint pain, immune boosting, gynecological disorders | Lihu, Guanxi, Yaozhai, Yaoshan | 0.3426 | HRC375 |
| *Sargentodoxa cuneata* (Oliver) Rehder & E. H. Wilson in Sargent | zhe ting lu | Sargentodoxaceae | liana | wild | stem | decoct in water and take orally | rheumatic bone pain, traumatic injury, amenorrhea in women, biliary ascariasis | Yaoshan | 0.0278 | HRC1199 |
|  |  |  |  |  |  | soak in wine and take internally | liver and kidney nourishment |  |  |  |
| *Saxifraga stolonifera* Curtis | e pi shou | Saxifragaceae | herb | wild | leaf | crush and apply topically | psoriasis | Lile, Huaili | 0.0648 | HRC241 |
|  |  |  |  |  |  | stir-fry with eggs | dysentery |  |  |  |
| *Schefflera heptaphylla* (Linnaeus) Frodin | tong tao ji | Araliaceae | tree | wild | root | soak in wine and take internally | rheumatic joint pain | Yaoshan, Guanxi | 0.0741 | HRC1231 |
|  |  |  |  |  | leaf | decoct in water and wash externally | athlete's foot |  |  |  |
|  |  |  |  |  | root | decoct in water and take orally | sore throat |  |  |  |
| *Schizocapsa plantaginea* Hance | fan ji mi | Dioscoreaceae | herb | wild | root | grind and apply externally | external injury bleeding | Guanxi, Yaoshan | 0.1204 | HRC1467 |
|  |  |  |  |  |  | decoct in water and take orally | stomach pain, whooping cough |  |  |  |
| *Secamone elliptica* R. Brown |  | Apocynaceae | liana | wild | whole plant | decoct in water and take orally / decoct in water and wash externally | rheumatism, bone pain | Yaozhai, Lile, Baha | 0.1481 | HRC195 |
| *Sedum sarmentosum* Bunge |  | Crassulaceae | herb | wild | whole plant | steam with lean pork | tuberculosis | Lile | 0.0185 | HRC237 |
| *Selaginella uncinata* (Desv.) Spring |  | Selaginellaceae | herb | wild | whole plant | decoct in water and take orally / decoct in water and wash externally | child high fever of unknown cause | Yaoli, Guanxi | 0.0741 | HRC35 |
| *Semiaquilegia adoxoides* (de Candolle) Makino | e ke | Ranunculaceae | herb | wild | tuber | decoct in water and take orally | hemorrhoids, asthma | Yaoshan, Dongjia, Huaili | 0.1389 | HRC1198 |
|  |  |  |  |  |  | crush and apply topically | snake bite, lymph node tuberculosis, furuncle infection |  |  |  |
|  |  |  |  |  |  | boil eggs（eat eggs） | lymph node tuberculosis |  |  |  |
| *Senecio scandens* Buchanan-Hamilton ex D. Don | e bei yi | Asteraceae | herb | wild | whole plant | decoct in water and take orally | clear heat and relieve fire | Yaozhai, Guanxi, Lile, Yaoli, Yaoshan | 0.3519 | HRC182 |
|  |  |  |  |  |  | decoct in water and wash externally | cataract, eye pain, body itch, hemiplegia, joint pain, gout |  |  |  |
| *Sigesbeckia orientalis* Linnaeus | e la kai | Asteraceae | herb | wild | tender leaf | take orally | diarrhea | Yaozhai | 0.0463 | HRC335 |
| *Sigesbeckia pubescens* (Makino) Makino | ji lu | Asteraceae | herb | wild | leaf | crush and apply topically | snake bite | Yaozhai, Dongjia | 0.0741 | HRC885 |
| *Sinobaijiania yunnanensis* (A. M. Lu & Zhi Y. Zhang) C. Jeffrey & W. J. de Wilde | zhu ai | Cucurbitaceae | liana | wild or cultivated | tuber | decoct in water and take orally | stomach pain, abdominal pain, acute necrotizing pancreatitis | Guanxi | 0.0278 | HRC291 |
| *Smilax biumbellata* T. Koyama |  | Smilacaceae | liana | wild | young stem sap | extract juice and apply externally | wound infection, remove necrotic tissue and promote granulation | Yaoli | 0.0185 | HRC439 |
| *Smilax china* Linnaeus | pa di | Smilacaceae | liana | wild | tender bud | crush and apply externally | scar removal | Yaoli, Yaoshan | 0.0741 | HRC59 |
|  |  |  |  |  | tuber | decoct in water and take orally | bone hyperplasia |  |  |  |
| *Smilax ocreata* A. de Candolle in A. de Candolle & C. de Candolle |  | Smilacaceae | liana | wild | sap from young stem | crush and apply externally | scar removal, remove necrotic tissue and promote granulation | Yaoli, Guanxi | 0.0556 | HRC61 |
| *Solanum americanum* Miller | yan jiu | Solanaceae | herb | wild | leaf | crush and apply topically | mouth ulcers, allergies | Guanxi | 0.0556 | HRC723 |
| *Solanum lyratum* Thunberg in Murray | ya gei | Solanaceae | herb | wild | whole plant | decoct in water and take orally | indigestion | Guanxi | 0.0278 | HRC710 |
| *Solanum nigrum* Linnaeus | e ka | Solanaceae | herb | wild | whole plant | crush and apply topically | unidentified swelling and infection, breast pain, scrofula, dental pain from heat | Yaoshan, Guanxi, Yaozhai | 0.0833 | HRC1428 |
|  |  |  |  |  |  | decoct in water and take orally | stomach cancer, esophageal cancer |  |  |  |
| *Solanum aculeatissimum* Jacquin |  | Solanaceae | herb | wild | root | chew and hold in mouth | gum swelling and pain | Yaoli, Lile, Yaoshan | 0.1667 | HRC85 |
|  |  |  |  |  |  | grind and take orally | sore throat |  |  |  |
|  |  |  |  |  |  | crush and apply topically | venomous snake bite |  |  |  |
| *Solidago decurrens* Loureiro | e bai | Asteraceae | herb | wild | whole plant | decoct in water and take orally | fever reduction, lung heat cough, sore throat, jaundice, eye inflammation | Yaoshan | 0.0463 | HRC1452 |
| *Sonerila cantonensis* Stapf | ya ge duo wu | Melastomataceae | herb | wild | root | decoct in water and take orally | diarrhea | Yaoli | 0.0278 | HRC40 |
| *Sophora tonkinensis* Gagnepain | shan dou gen | Fabaceae | shrub | wild | whole plant | decoct in water and take orally | antral gastritis, laryngeal cancer | Yaoli, Lile, Guanxi, Lihu, Dongjia | 0.4259 | HRC3 |
|  |  |  |  |  |  | take orally directly | abdominal pain |  |  |  |
| *Spiranthes sinensis* (Persoon) Ames | e wu hao | Orchidaceae | herb | wild | whole plant | stew with meat | coughing blood, physical weakness and emaciation | Yaoshan, Guanxi | 0.0556 | HRC1474 |
|  |  |  |  |  |  | decoct in water and take orally | sore throat |  |  |  |
|  |  |  |  |  |  | crush and apply topically | scald, snake bite, shingles |  |  |  |
| *Stachyurus chinensis* Franchet |  | Stachyuraceae | shrub | wild | whole plant | decoct in water and take orally | joint loosening, pain, urinary obstruction | Yaozhai, Lile, Guanxi | 0.1111 | HRC220 |
| *Stephania cephalantha* Hayata | jiu qu lao | Menispermaceae | liana | wild | tuber | crush and apply topically | traumatic injury, mild burn, abscess draining | Lile, Guanxi, Yaoshan | 0.1481 | HRC1203 |
| *Stephania dielsiana* Y. C. Wu | zhun zu wo | Menispermaceae | liana | wild | tuber | take orally directly | stomach pain, antral gastritis | Guanxi | 0.0370 | HRC388 |
| *Stephania japonica* (Thunb.) Miers |  | Menispermaceae | liana | wild | whole plant | decoct in water and take orally | abdominal pain | Lile, Baha, Guanxi | 0.1204 | HRC520 |
| *Stephania kwangsiensis* H. S. Lo |  | Menispermaceae | liana | wild | tuber | crush and apply topically | traumatic injury | Lile, Guanxi | 0.0833 | HRC329 |
|  |  |  |  |  |  | decoct in water and take orally | intestinal cancer, gastritis, weakness in hands and feet |  |  | HRC329 |
| *Stephania subpeltata* H. S. Lo |  | Menispermaceae | liana | wild | root | decoct in water and take orally | abdominal pain | Baha | 0.0185 | HRC540 |
| *Streptocaulon juventas* (Lour.) Merr. |  | Apocynaceae | liana | wild | root | decoct in water and take orally | abdominal pain | Guanxi | 0.0556 | HRC923 |
| *Striga asiatica* (Linnaeus) Kuntze | liao pu bi | Orobanchaceae | herb | wild | whole plant | decoct in water and take orally | child abdominal distension, child indigestion, cough, child fever | Yaoshan, Yaozhai, Guanxi, Yaoli | 0.2037 | HRC1431 |
| *Strobilanthes cusia* (Nees) Kuntze | yin zhei | Acanthaceae | herb | wild | leaf | crush and apply topically | mumps | Guanxi | 0.0278 | HRC446 |
| *Strobilanthes dimorphotricha* Hance | nong ce | Acanthaceae | herb | wild | whole plant | decoct in water and take orally | cough, pneumonia, rheumatism, bone pain | Yaoli | 0.0093 | HRC1 |
| Strobocalyx esculenta (Hemsley) H. Robinson et al. |  | Asteraceae | herb | wild | old stem, root | crush and apply topically | bone setting | Lile, Guanxi, Yaozhai | 0.0370 | HRC157 |
| *Taraxacum mongolicum* Hand.-Mazz. | e hai ka shou | Asteraceae | herb | wild | whole plant | crush and apply topically | unidentified swelling and infection | Yaozhai, Yaoshan | 0.1019 | HRC1444 |
|  |  |  |  |  |  | decoct in water and take orally | acute mastitis, gastritis |  |  |  |
| *Tetradium ruticarpum* (A. Jussieu) T. G. Hartley | mi la | Rutaceae | shrub | wild or cultivated | fruit | take orally directly | aid digestion | Huaili, Yaozhai, Guanxi, Baha | 0.2963 | HRC568 |
| *Tetrapanax papyrifer* (Hooker) K. Koch |  | Araliaceae | shrub | wild | root | decoct in water and wash externally | hemiplegia | Baha, Lile | 0.0833 | HRC228 |
|  |  |  |  |  |  | decoct in water and take orally | child constipation |  |  |  |
| *Tetrastigma planicaule* (J. D. Hooker) Gagnepain |  | Vitaceae | liana | wild | stem | soak in wine and take internally | stomach pain, joint pain, immune boosting | Lile, Guanxi | 0.1111 | HRC155 |
| *Tinospora sagittata* (Oliver) Gagnepain | zhei zhu gua | Menispermaceae | liana | wild | tuber | decoct in water and take orally | acute necrotizing pancreatitis | Guanxi, Yaozhai, Yaoli, Lihu, Yaoshan | 0.2685 | HRC276 |
| *Tinospora sinensis* (Loureiro) Merrill | zhuo song zhe ru | Menispermaceae | liana | wild | stem | soak in wine and take internally / soak in wine and apply externally | relax tendons and activate circulation | Guanxi, Yaozhai, Yaoli | 0.1296 | HRC412 |
| *Toddalia asiatica* (Linnaeus) Lamarck | jiu piao ca | Rutaceae | shrub | wild | root, old stem | soak in wine and take internally | immune boosting | Yaoshan, Dongjia, Lihu, Yaoli, Guanxi, Yaozhai, Baha, Lile | 0.4444 | HRC66 |
|  |  |  |  |  |  | soak in wine and apply externally | traumatic injury, internal injury, swelling reduction, rheumatic bone pain, joint pain |  |  |  |
|  |  |  |  |  |  | decoct in water and take orally | traumatic injury, internal bleeding, excessive menstruation in women |  |  |  |
| *Toona sinensis* (A. Jussieu) M. Roemer |  | Meliaceae | tree | cultivated | tender bud | decoct in water and take orally | white discharge turning yellow | Yaoli, Yaozhai | 0.0463 | HRC49 |
|  |  |  |  |  |  | crush and apply topically | snake venom antidote |  |  |  |
| *Toricellia angulata* Oliver | tong bao | Toricelliaceae | shrub | cultivated | root bark | crush, mix with alcohol, and apply externally | fracture | Yaoshan, Baha | 0.0278 | HRC1237 |
|  |  |  |  |  |  | soak in wine and take internally | rheumatism |  |  |  |
|  |  |  |  |  |  | steam with meat | amenorrhea in women |  |  |  |
| *Tradescantia pallida* (Rose) D. R. Hunt | zhu long jie | Commelinaceae | herb | wild | whole plant | crush and apply topically | vitiligo | Guanxi, Huaili | 0.0833 | HRC447 |
| *Tradescantia zebrina* Bosse |  | Commelinaceae | herb | wild | whole plant | saok in wine with other medicinal plants | immune boosting | Lile | 0.0463 | HRC161 |
| *Triadica sebifera* (Linnaeus) Small |  | Euphorbiaceae | tree | wild | leaf | crush and apply topically | snake bite | Baha | 0.0370 | HRC815 |
| *Trichosanthes rosthornii* Harms in Engler | jie liao | Cucurbitaceae | liana | wild | root | decoct in water and take orally | stomach pain, tuberculosis | Lile, Guanxi | 0.0648 | HRC141 |
| *Trichosanthes* sp. |  | Cucurbitaceae | liana | wild | tuber | grind, mix with water and take orally | erectile dysfunction, penile shrinkage | Lile | 0.0185 | HRC246 |
| *Trichosanthes truncata* C. B. Clarke in J. D. Hooker | guo ai | Cucurbitaceae | liana | wild | tuber | grind, mix with water and take orally | stomach pain, cancer | Guanxi | 0.0556 | HRC681 |
| *Triumfetta rhomboidea* Jacquin |  | Malvaceae | herb | wild | leaf | crush and apply topically | snake bite | Dongjia | 0.0278 | HRC884 |
| *Uncaria rhynchophylla* (Miquel) Miquel ex Haviland | zhu gong te | Rubiaceae | liana | wild | branches and leaves | soak in wine and take internally | high blood pressure, menstrual irregularities | Yaoli, Guanxi, Yaozhai | 0.1667 | HRC369 |
|  |  |  |  |  | stem | decoct in water and take orally | traumatic injury, activate blood circulation and remove stasis |  |  |  |
| *Urena lobata* Linnaeus | zhe ru | Malvaceae | shrub | wild | root | decoct in water and take orally | infectious jaundice, hepatitis, skin carbuncles | Yaoli | 0.0648 | HRC34 |
| *Urtica fissa* E. Pritzel |  | Urticaceae | herb | wild | whole plant | crush, mix with wine and apply topically | venomous snake bite | Dongjia, Huaili, Yaoshan, Guanxi, Lile | 0.2407 | HRC113 |
|  |  |  |  |  |  | decoct in water and wash externally | unblocking channels, paralysis, gonorrhea, syphilis |  |  |  |
|  |  |  |  |  |  | decoct in water and take orally | witchcraft poison antidote |  |  |  |
|  |  |  |  |  |  | steam with pork stomach | antral gastritis |  |  |  |
| *Vaccinium bracteatum* Thunb. | tong tu nao | Ericaceae | shrub | wild | leaf | medicinal tea | blurred vision, physical weakness | Yaoshan, Guanxi | 0.0926 | HRC1418 |
|  |  |  |  |  |  | crush, cook with eggs | diarrhea |  |  |  |
|  |  |  |  |  |  | decoct in water and wash externally | skin itching |  |  |  |
|  |  |  |  |  | root | decoct in water and wash externally | toothache, traumatic injury |  |  |  |
| *Verbena officinalis* Linnaeus | liao dao na rao | Verbenaceae | herb | wild | whole plant | decoct in water and take orally | cramps, fever reduction, fever, abdominal pain, stones | Huaili, Lile, Guanxi | 0.2037 | HRC131 |
| *Vernicia fordii* (Hemsley) Airy Shaw |  | Euphorbiaceae | tree | cultivated | juice from the immature fruit | extract juice and apply externally | furuncle, anti-itch | Guanxi | 0.0278 | HRC440 |
| *Veronica peregrina* Linnaeus | e qi yong | Plantaginaceae | herb | wild | whole plant | soak in wine and take internally | traumatic injury | Yaoshan | 0.0556 | HRC1432 |
|  |  |  |  |  |  | crush and apply topically | fracture |  |  |  |
|  |  |  |  |  |  | decoct in water and take orally | coughing blood, amenorrhea |  |  |  |
| *Viburnum fordiae* Hance |  | Adoxaceae | shrub | wild | leaf | mix with Triadica sebifera leaves, crush and apply topically on the affected area | snake bite | Baha | 0.0278 | HRC816 |
| *Viburnum utile* Hemsley | pi che chao | Adoxaceae | shrub | wild | root | decoct in water and take orally | dysentery, rheumatic pain | Yaoshan | 0.0556 | HRC1437 |
|  |  |  |  |  | leaf | crush and apply topically | external injury bleeding |  |  |  |
|  |  |  |  |  |  | stew with pork intestine | hemorrhoids, excessive vaginal discharge in women |  |  |  |
| *Vigna angularis* (Willdenow) Ohwi & H. Ohashi | dou qu la | Fabaceae | herb | cultivated | seed | decoct in water and take orally | edema, insufficient lactation postpartum | Yaoshan, Yaoli, Guanxi, Yaozhai | 0.2315 | HRC1219 |
|  |  |  |  |  |  | crush and apply topically | joint sprain |  |  |  |
| *Viola diffusa Gingins* in Candolle |  | Violaceae | herb | wild | whole plant | decoct in water and take orally | fever reduction | Guanxi | 0.0185 | HRC452 |
| *Viola fargesii* H. Boissieu | ya gong gao | Violaceae | herb | wild | whole plant | decoct in water and take orally | high fever | Guanxi | 0.0278 | HRC722 |
| *Viola japonica* Langsdorff ex Candolle |  | Violaceae | herb | wild | whole plant | crush with rocks and apply topically | osteomyelitis | Yaoli | 0.0556 | HRC71 |
|  |  |  |  |  |  | crush and apply topically | abscess |  |  |  |
| *Viola philippica* Cavanilles |  | Violaceae | herb | wild | whole plant | crush and apply topically | carbuncle | Huaili | 0.0648 | HRC593 |
| *Vitex negundo* Linnaeus |  | Lamiaceae | shrub | wild | stem | decoct in water and take orally / soak in wine and take internally | witchcraft poison antidote, traumatic injury, dysentery, athlete's foot | Huaili | 0.1481 | HRC606 |
| *Vitex quinata* (Loureiro) Williams | yi zhu nuo | Lamiaceae | tree | wild | stem | decoct in water and take orally / soak in wine and take internally | witchcraft poison antidote, traumatic injury, dysentery, athlete's foot | Huaili | 0.0278 | HRC605 |
| *Vitis balansana* Planchon in A. Candolle & C. Candolle | wu pu tao | Vitaceae | liana | wild | root | decoct in water and take orally | lower blood pressure | Yaoli | 0.0185 | HRC19 |
| *Wikstroemia indica* (Linnaeus) C. A. Meyer | zhua shou yao | Thymelaeaceae | shrub | wild | whole plant | soak in wine and take internally | internal injury | Yaoli, Yaozhai, Guanxi | 0.1204 | HRC181 |
|  |  |  |  |  |  | soak in wine and apply externally | traumatic injury |  |  |  |
| *Xanthium strumarium* Linnaeus | liao she | Asteraceae | herb | wild | fruit | decoct in water and wash externally | anti-itch, urticaria | Guanxi | 0.0463 | HRC715 |
|  |  |  |  |  | whole plant | decoct in water and take orally | dysentery, rhinitis, malaria |  |  |  |
| *Xanthium chinense* Mill. | liao she | Asteraceae | herb | wild | whole plant | decoct in water and wash externally | urticaria | Guanxi | 0.0185 | HRC714 |
| *Zanthoxylum armatum* Candolle | zuo lie | Rutaceae | shrub | wild | root bark | grind and hold in mouth | toothache | Yaozhai, Lile, Guanxi, Huaili | 0.4444 | HRC76 |
|  |  |  |  |  |  | use the thorn to draw blood | venomous snake bite |  |  |  |
|  |  |  |  |  |  | take orally directly / crush and apply topically | venomous snake bite |  |  |  |
| *Zanthoxylum dimorphophyllum* Hemsl. | huo li lu | Rutaceae | tree | wild | root | soak in wine and take internally | rheumatism, stomach gas pain | Yaoshan, Huaili, Yaoli, Dongjia, Guanxi, Lile | 0.3333 | HRC1225 |
|  |  |  |  |  |  | crush and apply topically | traumatic injury |  |  |  |
|  |  |  |  |  |  | mix with water and take orally | tooth decay pain |  |  |  |
| *Zingiber lingyunense* D. Fang |  | Zingiberaceae | herb | wild or cultivated | tuber | soak in wine and take internally / soak in wine and apply externally | traumatic injury | Lile | 0.0278 | HRC243 |
| *Zingiber striolatum* Diels |  | Zingiberaceae | herb | wild | whole plant | decoct in water and wash externally | unblocking channels, paralysis | Dongjia, Guanxi, Baha | 0.0463 | HRC890 |
